# Consequences of Eukaryotic Enhancer Architecture for Gene Expression Dynamics, Development, and Fitness

**DOI:** 10.1371/journal.pgen.1002364

**Published:** 2011-11-10

**Authors:** Michael Z. Ludwig, Manu, Ralf Kittler, Kevin P. White, Martin Kreitman

**Affiliations:** 1 Department of Ecology and Evolution, University of Chicago, Chicago, Illinois, United States of America; 2 Institute for Genomics and Systems Biology, University of Chicago, Chicago, Illinois, United States of America; 3 Department of Human Genetics, University of Chicago, Chicago, Illinois, United States of America; Fred Hutchinson Cancer Research Center, United States of America

## Abstract

The regulatory logic of time- and tissue-specific gene expression has mostly been dissected in the context of the smallest DNA fragments that, when isolated, recapitulate native expression in reporter assays. It is not known if the genomic sequences surrounding such fragments, often evolutionarily conserved, have any biological function or not. Using an enhancer of the *even-skipped* gene of *Drosophila* as a model, we investigate the functional significance of the genomic sequences surrounding empirically identified enhancers. A 480 bp long “minimal stripe element” is able to drive *even-skipped* expression in the second of seven stripes but is embedded in a larger region of 800 bp containing evolutionarily conserved binding sites for required transcription factors. To assess the overall fitness contribution made by these binding sites in the native genomic context, we employed a gene-replacement strategy in which whole-locus transgenes, capable of rescuing *even-skipped^-^* lethality to adulthood, were substituted for the native gene. The molecular phenotypes were characterized by tagging Even-skipped with a fluorescent protein and monitoring gene expression dynamics in living embryos. We used recombineering to excise the sequences surrounding the minimal enhancer and site-specific transgenesis to create co-isogenic strains differing only in their stripe 2 sequences. Remarkably, the flanking sequences were dispensable for viability, proving the sufficiency of the minimal element for biological function under normal conditions. These sequences are required for robustness to genetic and environmental perturbation instead. The mutant enhancers had measurable sex- and dose-dependent effects on viability. At the molecular level, the mutants showed a destabilization of stripe placement and improper activation of downstream genes. Finally, we demonstrate through live measurements that the peripheral sequences are required for temperature compensation. These results imply that seemingly redundant regulatory sequences beyond the minimal enhancer are necessary for robust gene expression and that “robustness” itself must be an evolved characteristic of the wild-type enhancer.

## Author Summary

In this study we provide evidence that eukaryotic enhancers contain regulatory sequences that provide robustness of gene expression to genetic and environmental perturbation. The regulatory logic of tissue-specific gene expression is encoded by compact non-coding enhancer sequences. We hypothesized that enhancers function not merely to turn genes “on” or “off” but to do so under the range of genetic and temperature conditions experienced by developing embryos. We tested this hypothesis using an enhancer of the *even-skipped* gene of *Drosophila* as a model. The enhancer is composed of a “minimal element,” capable of recapitulating native expression in reporter assays, and potentially redundant but evolutionarily-conserved sequences surrounding the minimal element. We assayed the functional impact of the peripheral sequences on development, from *in vivo* gene expression to adult viability, to show that they are required for optimal performance under temperature and X chromosome dosage perturbations. Our results suggest that the architecture of enhancers is adjusted by natural selection to ensure robust gene expression. Such adaptive fine-tuning may explain how enhancers experience rapid sequence divergence between closely related species while exhibiting functional conservation.

## Introduction

The genetic code, a simple one-dimensional vector of only four symbols, is decoded for the most part by molecular machinery that adheres to a strict grammar for translating genetic information into functional molecules. The specificity of the genetic code forms the conceptual basis for constructing “annotated” genomes, a major effort of the post-genome sequencing era.

But the majority of functional information in a genome does not reside in its transcribed compartments, where this strict grammar applies, but rather in the vast sea of “noncoding” sequences specifying the regulatory logic of gene expression. For these sequences, and in particular for the *cis*-regulatory elements (CRE) controlling eukaryotic gene expression, originally called enhancers [1], there is as yet no general agreement about how to define, much less identify the “functional” unit of eukaryotic gene regulation [2], [3], [4], [5], [6]. Enhancers generally contain multiple closely spaced target binding sites for several distinct transcription factors [7], [8], an attribute that can be exploited to identify enhancer sequences *in silico* [5], [9], [10], [11], [12], [13]. The best definition of a CRE, however, remains a time-tested functional one – the smallest piece of contiguous DNA that is capable of recapitulating a spatio-temporal pattern of native gene expression when placed in front of a promoter and reporter gene (typically β-galactosidase or GFP) and reintroduced into the organism from which the sequence was taken [7]. We will refer to these experimentally defined CRE's as “minimal” elements or enhancers.

Experimentally defined enhancers have discrete physical boundaries necessarily, but such discreteness is difficult to justify biologically. Sequences to which a transcription factor (TF) binds can only be described probabilistically [14], [15], owing to the fact that binding is not to unique target but rather to variants of a short sequence motif. TF binding occurs, therefore, not only at canonical or “high affinity” sites —ones that are typically identified in *in vitro* assays— but also to numerically abundant “low affinity” sequences [16], which are ever-present both within and beyond the margins of minimal enhancers. Evidence for their functionality comes primarily from modeling gene expression: weaker bioinformatically-identified sites are required to correctly predict activity [17], some taking the extreme approach of including all possible TF-DNA occupancy configurations [18], [19].

Furthermore, it is also not always possible to decompose the sequences contributing to activity in a tissue into discrete units. The expression in stripe 7 of the *Drosophila melanogaster even-skipped* (*eve*) gene receives contributions both from sequences in the “3+7” enhancer and from proximal sequences [17], [20], [21]. Similarly, the dorsal expression of *shavenbaby* in larval trichomes is derived from both the “E” and “7” enhancers [22] and both anterior and posterior *giant* (*gt*) domains receive input from multiple enhancers [23].

A lack of discrete CRE boundaries is also evident in the evolutionary analysis of binding site gain and loss, commonly referred to as “binding site turnover”. Comparing CRE sequences between the sibling species' *D. melanogaster* and *D. simulans* reveals the loss or gain of approximately 5% of transcription factor binding sites (TFBS) that have been functionally validated in well-characterized *D. melanogaster* enhancers [24], [25]. Binding site loss in *D. simulans* is expected to be offset by the gain of novel sites for the same TF, but this is not the case if the search for predicted new sites is restricted to the intervals identified as CREs in *D. melanogaster*. Additional novel binding sites are identifiable, however, if the search interval is expanded by 200bp in either direction, thus implying a functional role for these flanking sequences.

It is also not uncommon to find evolutionarily conserved instances of a TFBS beyond the borders of a minimal CRE, as exemplified by the *Drosophila eve* stripe 2 enhancer (S2E) [26], [27], the subject of this investigation. The *eve* minimal stripe 2 element (MSE), arguably the most intensively studied of all eukaryotic enhancers, constitutes a 480bp noncoding fragment located approximately 1000bp upstream of transcription initiation [20], [28] and contains a total of 12 “strong” TF binding sites, six sites for the activators Bicoid (Bcd) and Hunchback (Hb) and six sites for the repressors Krüppel (Kr) and Gt, as well as several additional “weak” Kr sites [29], [30]. *In vitro* DNAase footprinting experiments also identified three additional Kr binding sites beyond the borders of the minimal enhancer as well as two additional Hb sites [29]. But these binding sites appear to be redundant for S2E expression since the minimal element lacking them can direct a transverse band of expression in the blastoderm embryo at the same location as the native *eve* stripe 2 [31], [32], [33]. Comparative analysis of sequences across *Drosophila* phylogeny, however, indicates that all but possibly one site (hb-1) are conserved to the same extent as many sites internal to the MSE, indicating that they are functional [27].

The empirical definition of the enhancer itself, with discreteness as its conceptual underpinning, has yet to be put to experimental test. Accordingly, we ask here whether the MSE recovers the complete biological activity of the wildtype enhancer, that is, whether the conserved flanking binding sites are redundant or not.

Among the many biological characteristics of enhancer activity we could measure, we wished to investigate possible differences in the functional robustness of the MSE *versus* wildtype S2E for the following reason: an enhancer is expected to evolve not simply as a regulatory switch to turn gene expression on or off in response to a set of upstream signals, but also to do so “correctly” across the range of variability found in the signaling system. This variability is expected to arise by intrinsic molecular noise [34], [35], [36], [37], [38], [39], [40], [41], genetic variability in upstream factors and processes [42], [43], [44], as well as by external environmentally imposed conditions, such as the temperature-dependent rate of development [34], [45]. We hypothesize that certain features of enhancer architecture, such as the multiplicity of binding sites for a transcription factor, have evolved to assure stable enhancer performance rather than its switch-like behavior *per se.* Under this hypothesis, novel binding sites that make the enhancer more “robust” to perturbation will be selectively favored and will thus be incorporated into the enhancer architecture by a process of accretion [46]. The periphery of a CRE, we further hypothesized, might be a good place to look for the presence of such functional elements. Comparing the performance of the *eve* MSE *versus* the wildtype S2E would allow us to test this hypothesis.

To answer these questions we employed several experimental innovations. First, we investigated the temporal dynamics of stripe formation in individual live embryos, a characteristic of developmental robustness not observable with fixed, stained embryos. For this purpose, we created a fusion of the Eve protein with the fluorescent protein SYFP2, a fast-folding variant of the Yellow Fluorescent Protein (henceforth called YFP), to allow temporally resolved measurements of Eve stripe formation. A “live Eve” system also allowed us to synchronize the measurements across embryos to a specific nuclear division so that we could observe events in “wall-clock” time, independent of the status of developmental markers of pattern formation. The fusion protein additionally allowed us to measure not only Eve stripe 2 driven by either the MSE or the wildtype enhancer but all seven Eve stripes simultaneously. The additional stripe information internal to each embryo, genetically invariant landmarks surrounding stripe 2, allowed us not only to better characterize stripe 2 phenotypes, but also to investigate novel aspects of stripe formation dynamics. We could also investigate the robustness of enhancer performance in embryos developing at different growth temperatures.

Second, advances in transgene construction technology made it possible to investigate the S2E in a ∼16.4 kb transgene containing the entire native *eve* locus [26], [47]. Specifically, we investigated the *eve* locus (1) with the complete and unaltered S2E in its native context; (2) with the two regions flanking the MSE that contain the additional mapped Kr and Hb sites deleted, leaving behind the MSE sequence to drive eve stripe 2 expression; and (3) with an inverted version of the MSE to test the importance of enhancer orientation.

Third, we used site-directed transgenesis to place each version of the *eve* locus into the same target site in the *Drosophila* genome [48], [49]. All comparisons, therefore, were carried out in co-isogenic strains, eliminating any potential position effect of transgene insertion. Finally, genetic crosses allowed us to eliminate the functional native *eve* locus and to replace it with the whole-locus transgene copy. In doing so, we could investigate developmental phenotypes such as *engrailed* (*en*) expression, or fitness traits such as viability, in addition to molecular aspects of stripe formation. The ability to examine molecular, developmental, and organismal phenotypes with this novel experimental system allowed us to establish the molecular causality of defects occurring later in the life cycle of the fly, and therefore to establish a more complete biology of the MSE *versus* wildtype *eve* stripe 2 regulatory element.

## Results

**Figure 1:**
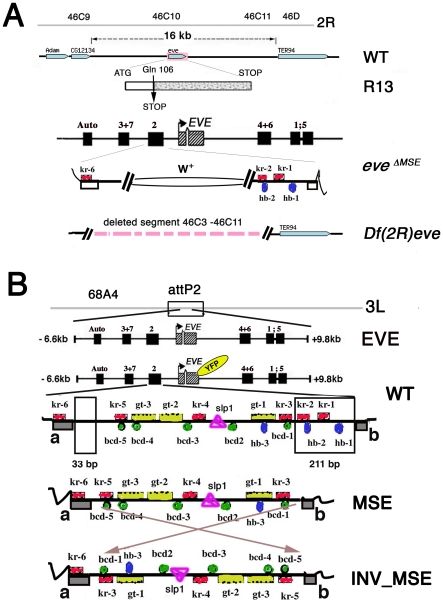
Strategy for creating co-isogenic strains carrying an *eve* locus with WT, MSE, and INV_MSE versions of the stripe 2 enhancer. A. Summary map of the *eve* locus and the three *eve* mutant alleles used in this study: the null alleles *eve^R13^* (R13) and *Df(2R)eve* (*Df(eve)*), and the MSE-deleted allele *eve^ΔMSE^*. CG12134 and TER94 are adjacent open reading frames. The late element (Auto) and early stripe enhancers are shown. In the *eve^ΔMSE^* lethal mutant, the 480-bp fragment corresponding to MSE was replaced by the *white^+^* gene by ends-out homologous recombination. Flanking *trans*-factor binding sites in the S2E: Kr (red squares) and Hb (blue ovals) are shown. Complete set of the binding sites in S2E is shown in panel B. B. Four versions of *eve* locus transgenes targeted to the attP2 docking site. The two regions deleted to create MSE and INV_MSE are highlighted in the WT S2E. Grey blocks a and b in WT are conserved sequences forming the two borders of the enhancer. The fluorescent tag is shown as YFP in yellow oval. Footprinted *trans*-factor binding sites in the S2E from *D. melanogaster* are shown: five Bcd (green circles), three Hb (blue ovals), six Kr (red squares), three Gt (yellow rectangles), and one Sloppy-paired1 (pink triangle) binding site. The numbers of the binding site are as in [29]. Sequences are shown in Text S1.

Our strategy was to create co-isogenic lines differing only in the S2E sequence carried by the transgenic *eve* locus. Three versions of the *eve-YFP* transgene were constructed: WT, which contains the “wildtype” reference stripe 2 enhancer sequence; MSE, which has a truncated stripe 2 enhancer; and INV_MSE, which has the orientation of the truncated stripe 2 enhancer inverted (Figure 1). Co-isogenic strains were created by targeting transgene insertion to the attP2 docking site. As we will show in the following section, a wildtype *eve* locus transgene lacking the YFP fusion, targeted to the attP2 site, delivers nearly full adult viability when it replaces the native *eve* locus.

The results are presented in three sections. The first section addresses a fundamental biological question: Do the MSE and INV_MSE versions of the transgene make a viable fly (*e.g.*, are they less fit than WT)? To measure *eve* transgene viability, we eliminated the native *eve* locus by crossing the transgene into one of three *eve* mutant backgrounds: *eve^R13^* (R13), a null allele created by a coding point mutation; Df(2R)*eve* (*Df(eve)*), a small deletion covering the entire *eve* locus and its immediate neighbors, and *eve^ΔMSE^*, the native locus in which the MSE region of the stripe 2 enhancer is deleted and replaced with *w^+^* sequence (Figure 1A). The recovery of complete wildtype viability when only transgenic *eve* is available is a stringent test of the suitability of the target site and whole-locus transgene for the proposed experiments. Next we compare the viability of WT, which encodes the Eve-YFP fusion, to that of the unmodified *eve* transgene to test the YFP tag's effect on the panoply of *eve* function. The recovery of good viability in WT permitted us to investigate the extent to which adult viability is compromised when *eve* stripe 2 expression is driven by either MSE or INV_MSE.

Section two reports the effects of WT, MSE, and INV_MSE on Engrailed patterning in an attempt to link differences in the viability in the two MSE genotypes compared to WT to Eve stripe-2-specific segmentation defects.

Having established this link, we present in the third section a detailed analysis and comparison of *in vivo eve* stripe 2 expression phenotypes. We first present experimental results that compare Eve-YFP and Eve expression to establish the fidelity of the Eve-YFP reporter. This is followed by a detailed analysis of Eve stripe 2 initiation, maturation and precision of border placement in WT, MSE, and INV_MSE. The section closes with an analysis of the response of stripe 2 expression phenotypes to embryonic growth temperature.

### Viability

**Figure 2:**
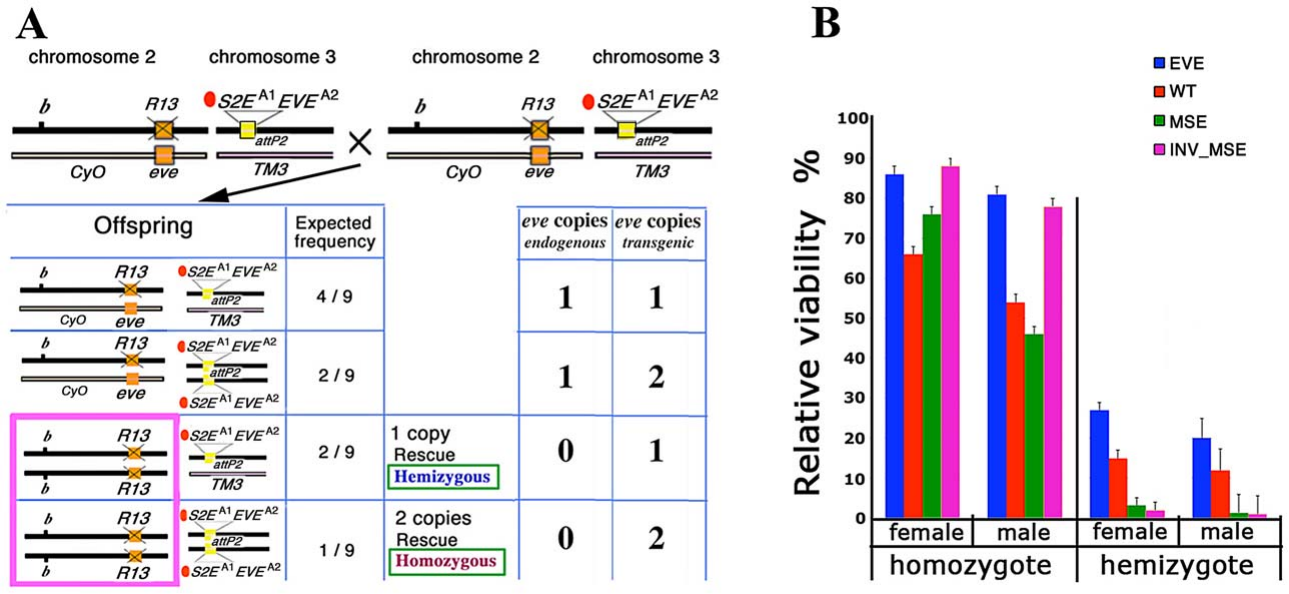
Relative viability: rescue of R13 lethality by the four studied transgenes. A. Schema for estimating adult viability. Example cross and relevant offspring genotypes for the viability assay (see Methods for details). Genetic notation — *CyO* and *TM3* are the second and third chromosome balancers respectively; *b:* mutant allele of *black;* orange box: native *eve;* R13 and X'd out orange box: R13 lethal mutant; attP2 docking site; yellow box: transgene attP2[*S2E^A1^EVE^A2^*]. A1 indicates the allele of S2E used (wt, MSE, or INV_MSE) and A2 indicates the allele of *eve* coding sequence used (wt or YFP). For each offspring genotype, the expected frequency and the number of endogenous and transgenic *eve* loci copies are shown. The hemizygous and homozygous rescue genotypes are highlighted in the pink rectangle. B. Relative viability is shown as rescue percentages for four studied transgenes: EVE (blue), WT (red), MSE (green), and INV_MSE (pink). EVE: attP2[*S2E^wt^EVE^wt^*], WT: attP2[*S2E^wt^EVE^YFP^*], MSE: attP2[*S2E^MSE^EVE^YFP^*], and INV_MSE: attP2[*S2E^INV_MSE^EVE^YFP^*]. The reduced viability of the hemizygous genotypes relative to homozygous genotypes (haploinsufficiency) is observed for all transgenes (Tables S1 and S2). The rescue percentages of EVE and the other three transgenes were evaluated in two separate experiments (Tables S1 and S2). Note the low rescue potency of homozygous MSE males. Error bars are binomial standard deviations.

To avoid complications of position effect on gene expression, all the studied transgenes were integrated into the same chromosome 3 attP2 docking site (3L [68A4]) [49]. To establish the suitability of this target site for expressing *eve,* we created a fly line, attP2[*S2E^wt^EVE^wt^*] (henceforth called EVE), in which the 16.4 kb unmodified native *eve* locus, *sans* a *YFP* tag or any modification to the S2E, was integrated into the target site. We evaluated the ability of this transgene to restore egg-adult viability when crossed into an Eve null (R13) genetic background (Figure 2A; Table S1).

The wildtype *eve* transgene (EVE) yielded very high homozygous rescue percentages (80 – 85%)—more than twice the corresponding rescue percentages previously reported for a slightly smaller version of the transgene [26], [47], [50] (also Methods)— that were not significantly different from full 100% rescue (*p>0.5*; Figure 2B; Table S1). Unless otherwise stated, all statistical testing for viability used the chi-squared test. The improvement in rescue ability may be due either to properties of the attP2 target site and/or to the inclusion of an additional sequence at the 3′ end of the transgene that contains insulator binding sites (Methods). Hemizygous rescue percentages are lower than for homozygotes, as expected for this haplo-insufficient locus [50], [51]. No significant difference was observed in the recovery of males *versus* females for either the hemizygous (*p = *0.43) or homozygous (*p = *0.88) rescue classes, a point to which we return in the following section.

#### Impact of YFP tag on fly viability

To monitor Eve stripe formation, we constructed the real-time 4D reporter *eve* transgene, WT (attP2[*S2E^wt^EVE^YFP^*]), by fusing a YFP-encoding sequence to the C-terminal of the *eve* coding region (Figure 1B). We first investigated the impact of the YFP tag in WT on egg-adult viability, a sensitive biological indicator of the fusion protein's effect on Eve protein function. The estimated rescue percentages of WT were consistently lower than for the corresponding *eve* transgene lacking the YFP tag (Figure 2B, Tables S1 and S2), but a robust proportion of adults of both homozygous and hemizygous genotypes were recovered. Therefore, although the YFP tag does reduce lifetime fitness, its overall functional impact is modest.

#### Minimal stripe enhancers make a viable fly

Having established the functionality of the fusion transgene (WT), we proceeded to investigate the impact of removing sequences in the stripe 2 enhancer region to create two “minimal” stripe 2 enhancers, MSE (attP2[*S2E^MSE^EVE^YFP^*]) and INV_MSE (attP2[*S2E^INV_MSE^EVE^YFP^*]). Can the *eve* locus with a total of 244bp of DNA deleted from the stripe 2 enhancer region, eliminating two footprinted binding sites each for the transcription factors Kr and Hb (kr-2, kr-1, hb-2 and hb-1), make a sufficiently functional stripe to produce a viable fly?

To our surprise, flies homozygous for either MSE or INV_MSE in an R13 (native *eve* null) background were both highly viable (Figure 2B) and fertile (data not shown). MSE relative viability was not significantly reduced compared to WT for either sex (*p(males)  = 0.28*; *p(females)  =  0.36*). INV_MSE homozygotes also did not exhibit reduced survival, and in fact showed significantly higher survival than WT (*p(males) < 0.005*; *p(females)  =  0.01*).

Two conclusions can be drawn from these observations. First, the fact that the MSE transgene, the smallest DNA fragment capable of recapitulating a stripe 2 in a reporter assay, is sufficient to produce a biologically functional Eve stripe, provides a strong validation of the empirical reporter approach to identifying functional units of *cis-*regulatory DNA. Moreover, the fact that INV_MSE is also biologically active confirms the S2E's conformity to orientation independent functionality, a fundamental principle of enhancer activity [1]. Second, physically mapped and evolutionarily conserved transcription factor binding sites for Kr and Hb that are deleted in MSE are not required for the enhancer to function biologically. Sequence conservation, in this context, does not equate to functional essentiality.

Although MSE and INV_MSE restores full viability when homozygous, it might be argued that this viability is not so much a function of the minimal enhancers delivering wildtype activity but rather the ability of the developmental system to buffer against variation in Eve stripe 2 expression. To test this hypothesis, we investigated the ability of MSE and INV_MSE to restore viability under more challenging conditions — when only one copy of the transgene was present in a fly. Strongly supporting the hypothesis, hemizygous MSE or INV_MSE exhibited strongly reduced adult viability, averaging less than 5%, as compared to ∼30% for WT (Figure 2B, Table S2). This relative reduction in viability must be attributable to functional differences between the MSE and WT versions of the S2E.

#### Sex ratio

The viability experiments allowed us to compare males and females of a given genotype, and we observed a distortion of the sex ratio favoring females in all genotypic classes and all transgenes (Figure 2B and Table S2), including genotypes carrying a copy of the native *eve* locus. Of particular interest, however, is a greater relative loss of male viability in MSE homozygotes as compared to WT (*p = 0.01*), suggesting the possibility of a functional difference in MSE-driven stripe formation between males and females. A similar, and even more severe, trend was seen for the small number of MSE hemizygote survivors compared to WT.

To further investigate this surprising sex-dependent viability loss, we carried out a cross (Figure S1) that is expected to yield 50% hemizygotes (compared to 1/9^th^ in the standard viability assay; see Figure 2A). We again observed a reduction in the viability of hemizygous MSE and INV_MSE compared to WT, but more importantly a 4-6-fold reduction in the recovery of males compared to females (Figure S1, *p = 2.3E-23* and *p = 1.3E-11*, respectively). Two additional crosses further confirmed the sex-dependent viability difference in MSE genotypes. In these crosses the native *eve* locus was eliminated not with the R13 *eve* null allele, but with either *Df(eve)* or *eve^ΔMSE^* (Figure 1 and Methods). In both crosses, the number of hemizygous males relative to females was reduced in MSE and INV_MSE compared to WT (Figures S2 and S3). The cross with *eve^ΔMSE^* (Figure S3) was particularly informative in this regard. *eve^ΔMSE^*/R13 offspring were diploid for all *eve cis*-regulatory regions except for the S2E — for which they were haploid. In this case the rescue by the WT S2E allele showed no sex bias, whereas the MSE and INV_MSE-rescued males had significantly lower viability (Figure S3, *p = 2.1E-5* and *p = 0.012*, respectively). Similarly, R13/*CyO* offspring, which were diploid for the regions flanking the minimal enhancer in the WT rescue but haploid in the MSE or INV_MSE rescues, also had significantly lower male viability in MSE and INV_MSE compared to WT (*p = 2.2E-5* and *p = 0.003*, respectively). Therefore for this cross, sex-dependent viability can be specifically attributed to the hemizygosity of the stripe 2 enhancer region in MSE and INV_MSE.

### Engrailed patterning

As the reduction in viability of the two hemizygous MSE genotypes (relative to WT*)* is due only to differences in the enhancer, we can make two predictions: (1) there will be developmental defects in the formation of *eve* stripe 2-dependent segments in MSE hemizygotes, and (2) these segmentation defects will create lethality at the embryonic stage. To test the latter prediction, we investigated embryo hatching rates from a cross expected to yield 50% hemizygotes in the rescue of R13 by the transgenes (Figure S4). The balancer chromosome was marked with Deformed-YFP (Dfd-YFP) and we measured relative viability by counting Dfd-YFP positive and negative hatched first instar larvae. Under the assumption that the heterozygous genotype *w; b,*R13*/CyO,p[Dfd-YFP];* attP2[*S2E^A1^EVE^YFP^*] has the same relative viability for all three versions the *eve* transgene (supporting evidence in Methods), prediction (2) can be tested by comparing the proportions of hemizygous WT *versus* MSE (or INV_MSE) transgene survivors (homozygous for R13). Consistent with this prediction, WT exhibited significantly better viability at this stage of development then either MSE or INV_MSE.

**Figure 3:**
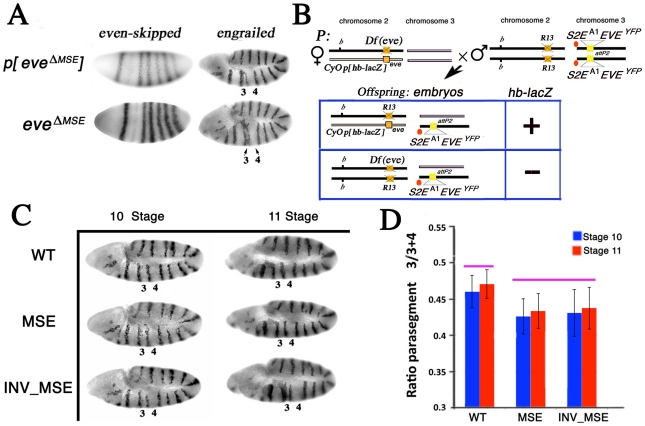
Effect of altered S2Es on Engrailed expression. A. *eve* mRNA and En protein expression patterns in stage 5 (blastoderm) and 10 embryos respectively. *p[eve^ΔMSE^]* transgene (*eve −6.4* to +8.4 kb without the 480 bp MSE) [26] and *eve^ΔMSE^* (native locus, where the 480bp MSE fragment was replaced by the *white^+^* gene; see Methods) drive qualitatively similar patterns of *eve* or En expression. Parasegments three and four in the En pattern are labeled as 3 and 4 respectively. B. Schema to study the En pattern in R13/*Df(eve)* mutant embryos rescued by the altered *eve* transgenes. Example cross and relevant offspring genotypes for the assay (see Methods for details). Genetic notation is as in Figure 2 except that the second chromosome balancer is marked with the lacZ gene driven by the *hb* promoter (*p[hb-lacZ]*). R13/*Df(eve)* mutant embryos having only one copy of the transgene are identifiable by the absence of β-galactosidase expression and PCR genotype. C. The En pattern in R13/*Df(eve)* mutant embryos having only one copy of the transgene WT, MSE, or INV_MSE. Stages 10 and 11. Note the variation in parasegments 3 and 4. D. Difference between WT and MSE (or INV_MSE) in En stripe 4 spatial expression was evaluated as a ratio of parasegment 3 length to the sum of the lengths of parasegments 3 and 4 (see Methods and Figure S6). Pink bars above the histogram mark genotypes and samples that do not differ significantly. Error bars are standard deviations. N is about 15 for each bar.

By confirming viability loss at the embryonic stage in MSE and INV_MSE hemizygotes, we proceeded to investigate whether specific defects in segmentation could be observed between the WT, MSE, and INV_MSE. The establishment of the *en* 14-stripe segment polarity gene expression pattern is a complex process that includes activation by *eve* early stripes [50]. Eve stripe 2 corresponds to parasegment 3, which is bordered by *en* stripes 3 and 4. These En stripes are developmental indicators of Eve stripe 2 expression: homozygous *eve^ΔMSE^* embryos lacking a functional stripe 2 enhancer produce a short parasegment three due to an anterior-shifted and vestigial En stripe 4 (Figure 3A). As expected, *eve^ΔMSE^* is a recessive embryonic lethal (Figure S5).

We focused attention on the position of En stripe 4 in stage 10 and 11 embryos by measuring its location relative to bordering stripes 3 and 5 (see Methods) in hemizygous embryos (Figure 3B, 3C). Both MSE genotypes exhibited a statistically significant reduction of parasegment 3 (*p<0.01*) when measured in embryos in which the native *eve* locus was removed (*Df(eve)/*R13) (Figure 3D; Table S3). We also investigated the same genotypes in stage 11 embryos when only the stripe 2 enhancer was deleted from the native *eve* locus (*eve^ΔMSE^ /*R13) and again found a significant reduction of parasegment 3 in MSE (*p<0.01*, the Mann-Whitney Wilcoxon ranksum test was used here and in all subsequent statistical testing) and nearly significant (*p = 0.08*) in INV_MSE hemizygotes (Figure S7; Table S4). The segmental defect revealed by En patterning in hemizygous embryos, therefore, can be attributed specifically to hemizygosity of MSE or INV_MSE.

### Dynamics of *eve* stripe 2 expression

The studies of adult viability and En patterning in MSE *versus* WT, as presented above, establish biological differences that are directly attributable to the structural differences in the enhancer itself. In the following sections, we investigate the dynamics of Eve stripe 2 formation and maturation, taking advantage of the ability to track its evolution in an individual embryo through time.

Differences in En patterning in WT and MSE could be the result of average differences in Eve stripe 2 patterning or greater variation among individual embryos. Analysis of stripe patterning in live embryos allowed us to investigate both features of stripe dynamics. Because the measurements of stripe formation and maturation involves data collected from live embryos, a brief overview of the methods employed and their validation is presented.

#### Validation of Eve-YFP expression in fixed tissue

In order for *eve-YFP* to be useful as a live reporter, it must express Eve-YFP protein with a high fidelity to the endogenous Eve pattern in space and time. In an approach similar to the one taken for validating *bcd-GFP* expression [52], we tested the fidelity of the Eve-YFP pattern at nuclear resolution by co-staining embryos carrying the *eve-YFP* transgene with anti-Eve and anti-GFP antibodies and measuring the mean fluorescence in each nucleus (see Methods). As shown in Figure S8A, fluorescence in the two channels exhibits a strongly linear relationship. Eve provides positional information in the embryo, and the correct positioning of its borders is essential for normal segmentation [53]. Due to their functional importance, we measured the border positions and found that Eve-YFP forms its borders in the same positions as endogenous Eve (Figure S8D).

Despite strongly proportional expression and identical border positions, closer inspection of overlays of anti-GFP and anti-Eve profiles from the same embryo shows that the Eve-YFP pattern exhibits a temporal lag compared to endogenous Eve (Figure S8B). The lag is less than 6 min since the expression profiles score in the same time class of the staging scheme of Surkova *et al*. [36]. This delay is perhaps not surprising since the fusion gene is longer than *eve* and would take more time to be transcribed and translated. The Eve-YFP pattern in fixed tissue shows the same WT, MSE and INV_MSE phenotypes as the live data (Figure S9).

#### Imaging of Eve-YFP expression in vivo

Since gene expression movies are acquired from each embryo in a separate experiment, we adopted an experimental methodology to ensure that data from individual embryos were comparable (see Methods). One of the strengths of live imaging is the ability to follow gene expression in absolute time without relying on the pattern of Eve maturation or embryo morphology to determine the age of an embryo. Instead, we registered the time series of embryos imaged in separate experiments by starting the clock at the completion of the thirteenth nuclear division, made possible by the presence of a *His2Av-RFP* transgene to mark nuclei, and were able to determine the age of each embryo relative to the starting point with a precision of 0.5 min (see Methods).

#### Quantitative Eve-YFP data from live embryos

**Figure 4:**
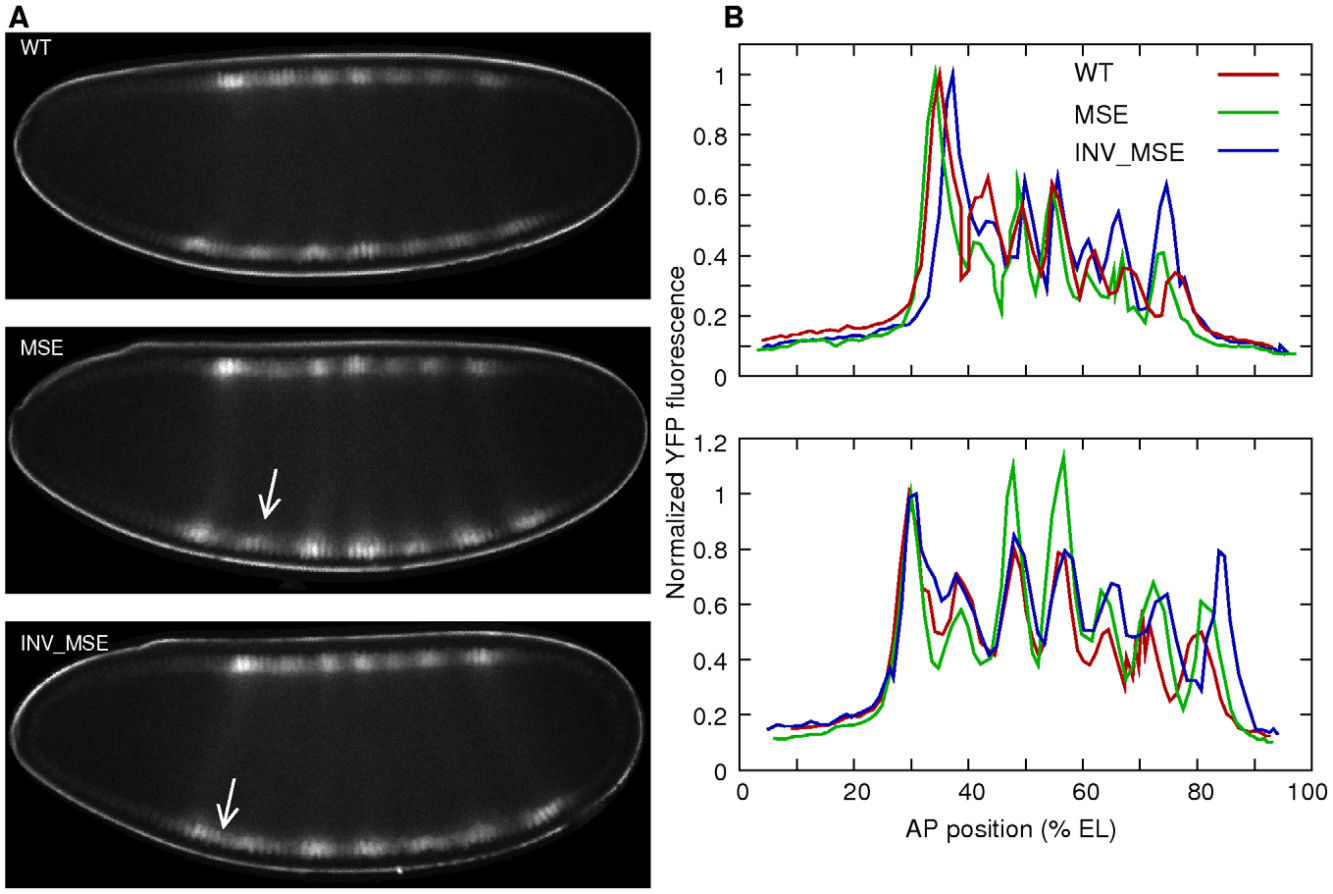
Eve-YFP expression in WT, MSE, and INV_MSE embryos. A. Sample embryos expressing Eve-YFP just prior to gastrulation. The expression patterns correspond roughly to mid cycle 14 Eve expression observed in fixed tissue [36], implying that the time required for YFP maturation induces a 15–20 min delay. Stripe 2 is weaker in MSE and the anterior border is derepressed in INV_MSE (arrows). Anterior is to the left and dorsal is up. B. Raw quantitative Eve-YFP expression data extracted from the dorsal (top) and ventral (bottom) nuclei of the images shown in panel A. The *y*-axis is the mean fluorescence intensity in a nucleus and the *x*-axis is the anteroposterior (AP) position of the nucleus. The expression values are normalized to the peak of stripe 1. Both dorsal and ventral profiles show the mutant phenotypes. The ventral profiles exhibit a more mature Eve pattern than the dorsal ones. The other stripes are brighter relative to the first, and stripes 5 and 6 are more resolved. Anterior is 0% egg length (EL).

We used image segmentation techniques to automatically identify nuclei in the His2Av-RFP images and calculated the mean fluorescence intensity of Eve-YFP expression in the pixels lying inside each nucleus (Figure 4, see Methods). Eve-YFP profiles from individual embryos follow the same overall temporal progression as the one observed in fixed tissue (Video S1, [36], [54]. Despite general agreement with the chronology of Eve pattern maturation, the *in vivo* Eve-YFP pattern appears to lag behind that of endogenous Eve. Using the staging scheme of Surkova *et al*. [36], the live Eve-YFP patterns score in a time class occurring 15–20 min before the actual age of the embryo (legend of Figure 4A). This delay is much larger than the one observed between fixed-tissue Eve-YFP and Eve profiles. This is not surprising since fluorescent proteins require additional steps after folding to achieve fluorescence [55]. Because the temporal progression of *in vivo* Eve-YFP expression follows that of endogenous Eve, the main limitation resulting from this lag is that we cannot observe the most mature Eve expression having equally bright stripes [36] seen in the last quarter of cycle 14.

#### Phenotypes of Eve stripe maturation and initiation

**Figure 5:**
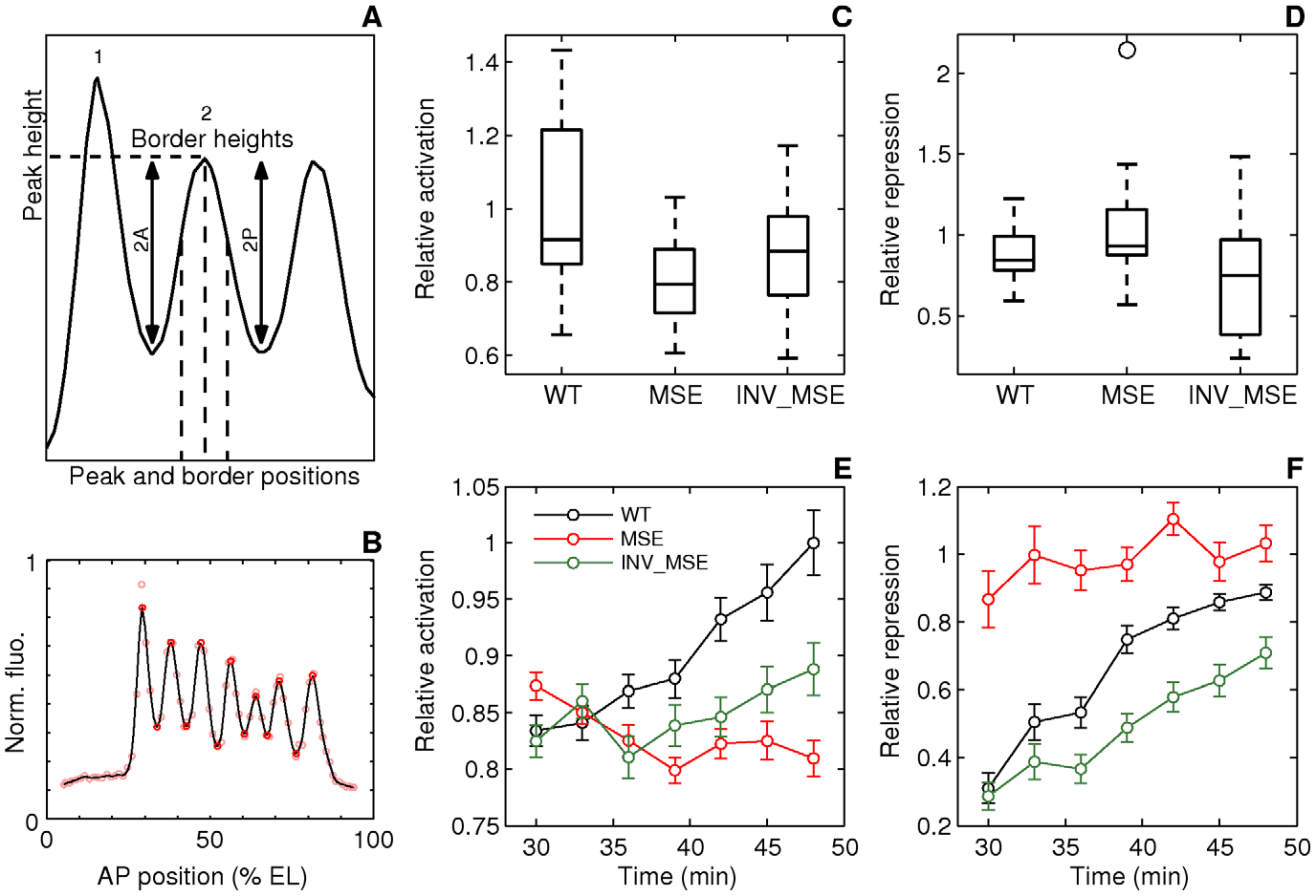
Dynamics of stripe 2 activation and repression. A. Schematic of the measured stripe 2 phenotypes. The black curve is an Eve expression profile showing the first three stripes. The *y*-axis is the relative fluorescence intensity and the *x*-axis is AP position. The horizontal dashed line is the fluorescence intensity at the peak. 2A and 2P are the heights of the anterior and posterior borders respectively, calculated as the difference between the intensities at the maximum and the associated minimum. The vertical dashed lines are positions of, from left to right, the anterior border, the peak, and the posterior border. B. The measurement of the features of the Eve expression pattern. The *y*-axis is fluorescence intensity normalized to values between 0 and 1. The pink circles are average Eve-YFP fluorescence intensities in individual ventral nuclei. The black line is a smoothing cubic spline fit to data (see Methods). The red circles are the local maxima and minima of the spline. Border position was measured as the position where the spline crosses the mean value of consecutively-occurring minima and maxima. C,D. Boxplots of relative activation and repression at 48 min. The box lines are the first quartile, median, and the third quartile. The whiskers extend to the most extreme values lying within 1.5 times the interquartile range and any datapoints outside the whiskers are shown as circles. C. Relative activation, measured as the ratio of the peak expression of stripes two and one, is lower in MSE (*p = 0.0114*). D. Relative repression, measured as the ratio of the heights of the anterior and posterior border, is lower but not statistically significant in INV_MSE (*p = 0.1321*) at this timepoint. N = 15, 15, and 14 for WT, MSE, and INV_MSE respectively. E,F. Time series of relative activation and repression. Time is measured from the completion of the thirteenth nuclear division. The lines show the means and the error bars are standard errors of the mean. The series start at 30 min, when most embryos have an incipient stripe 2 (Figure 6A). The data for each timepoint are extracted from the same sample of embryos. The same data underlie the boxplots in panels C and D and the 48 min timepoint. N is between 12-15, 14-19, and 9-15 for WT, MSE, and INV_MSE respectively. The sample size varies from timepoint to timepoint due to either heterochrony in the appearance of stripe 2 or occasional failure of the segmentation algorithm at some timepoints of an embryo. E. Relative activation. WT, MSE, and INV_MSE have the same relative expression levels initially. The WT stripe 2 increases expression over time but MSE fails to do so. Between WT and MSE, *p = 0.3380, 0.8159, 0.3286, 0.0836, 0.0611, 0.0577, 0.0114* for *t = 30–48* min. INV_MSE is intermediate. Between WT and INV_MSE, *p = 0.6985, 0.9770, 0.4233, 0.5972, 0.1249, 0.2808, 0.2300* for *t = 30–48* min. The lowered and intermediate activation of MSE and INV_MSE respectively do not depend on the stripe chosen for normalization (Figure S14A, S14C). F. Relative repression. WT and INV_MSE both have derepressed anterior borders initially while MSE has symmetric anterior and posterior borders (relative repression ∼ 1). The WT anterior border gets repressed over time to give almost symmetric borders prior to gastrulation. Between WT and MSE, *p = 0.0034, 0.0118, 0.0134, 0.1502, 0.0407, 0.3028, 0.1711* for *t = 30–48* min. The INV_MSE anterior border fails to get fully repressed. Between WT and INV_MSE, *p = 0.8603, 0.3408, 0.1821, 0.0565, 0.0620, 0.0815, 0.1321* for *t = 30–48* min. During the formation of the stripes, some embryos have very large border height ratios as a border is first established. Such values have been excluded from the time series plots to show the detail of the rest of the datapoints, but were included in all statistical testing.

Several Eve stripe 2 phenotypes could be measured in the live-image data. We extracted Eve-YFP profiles from the dorsal and ventral edge of each embryo by projecting onto the anteroposterior (AP) axis. Both profiles exhibit the same phenotypes (Figure 4B), but we restricted further analysis to ventral profiles only as they display the most mature Eve expression. Eve stripe 2 can be described by six features - expression level at the peak, the heights of the two borders, and the positions of the peak and borders (Figure 5A) - that can be detected using a spline approximation (Figure 5B and Methods).

**Figure 6:**
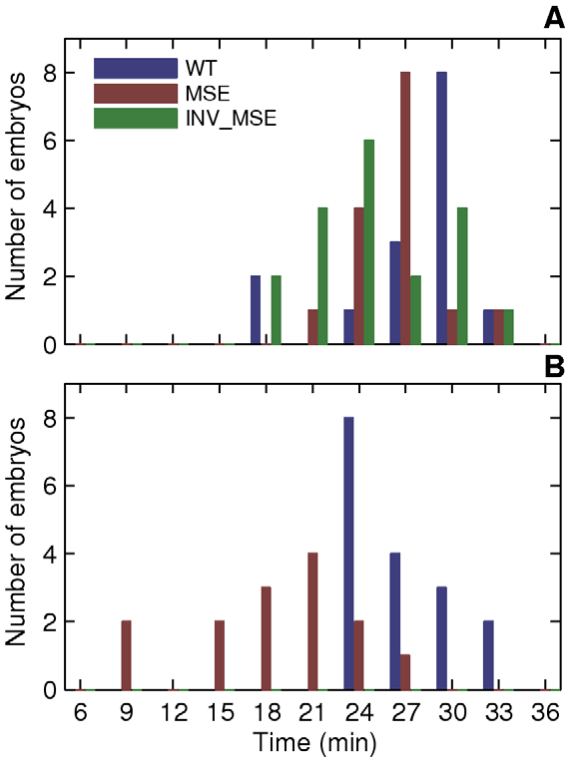
Initial appearance of an incipient stripe 2. We considered an incipient stripe 2 to have formed when both local minima corresponding to the future 1-2 and 2-3 interstripes were detected by the spline approximation (see Methods) and stably maintained at all subsequent time points. In some MSE embryos, stripe 2 was detectable at all but one future timepoints. Since the local minima were detected at all other timepoints, this is in all probability due to weak stripe 2 expression and not an error in detecting the incipient stripe. A. Histogram of the time of initial appearance of stripe 2 in WT, MSE, and INV_MSE at 25C. B. 29C, WT and MSE only.

One of the important added values of the *eve-YFP* fusion in a whole-locus transgene is that all seven Eve stripes are visible in the developing embryos, whereas only stripe 2 varies genetically. This allows us to scale the properties of stripe 2 relative to those of stripe 1, which serves as a within-embryo control on embryo-to-embryo experimental variation. Accordingly, we define two Eve maturation phenotypes, relative activation and relative repression, as the ratio of peak stripe 2 expression to that of stripe 1 and the ratio of the heights of the anterior and posterior borders respectively. The former is a measure of stripe 2 activation, while the latter is a measure of the repression of the anterior border. We present these measurements at three-minute intervals for a total of 18 minutes, from when stripe 2 is first detectable until gastrulation. Inspection of these temporally resolved data allow us to analyze not only the differences between WT and MSE at comparable developmental stages, but also differences in the temporal dynamics of stripe formation.

In addition to these stripe 2 maturation phenotypes, we also used the *in vivo* movies to investigate two additional phenotypes relating to the appearance of a nascent stripe.

#### Eve stripe 2 phenotypes at the late cellular blastoderm stage

We first describe stripe 2 differences at the late blastoderm stage just prior to the initiation of gastrulation. The “mature” Eve stripes produced by MSE and INV_MSE exhibit distinct defects compared to WT (Figure 4 and Figure 5C, 5D). The defects involve peak and border heights but not border positions, which did not change significantly (Figure S10). Although we observed embryo-to-embryo variation —this despite scaling stripe phenotypes relative to adjacent stripes in the same embryo— the MSE produces a “weaker” stripe 2 than either WT or INV_MSE, as evidenced by a comparison of relative activation (Figure 5C, *p = 0.0114)*. The INV_MSE appears to have a stripe of intermediate height.

At the late blastoderm stage, MSE and WT both produce a well-differentiated stripe: nearly identical values of relative repression signify equal repression of the anterior and posterior borders (Figure 5D). In contrast, INV_MSE appears to have a derepressed anterior stripe 2 border since it has lower values of relative repression (Figure 5D). Although the WT and INV_MSE distributions are not significantly different at this timepoint (*p = 0.1321*), the temporal dynamics of INV_MSE relative repression support the conclusion that the anterior border is derepressed (see below). These distinct molecular defects belie the similarities between the two minimal enhancers with respect to both viability and En patterning. Although MSE and INV_MSE are both functional, they are not entirely orientation independent. More careful analysis of the temporal dynamics of stripe formation, described below, reinforces this conclusion.

#### Dynamics of stripe maturation

Differences in the molecular phenotypes of MSE and INV_MSE compared to WT in late blastoderm embryos can be better understood from the dynamics of stripe formation. Imaging of stripes at three-minute temporal resolution during the time when eve stripe maturation occurs, coupled with the ability to synchronize the embryos to the same absolute time-clock, allowed us to investigate how the “mature” eve stripe phenotypes arose (Figure 5E, 5F). Initially, at the first appearance of an individualized stripe 2 (approximately 30 min.), the three enhancers show similarly low relative activation. The distributions of initial appearance times of a discernable stripe are overlapping for the three genotypes at 25°C (Figure 6A, *p = 0.0558* and *p = 0.0875*, when WT is compared to MSE and INV_MSE respectively). This early similarity in stripe 2 morphology suggests that initial Eve stripe 2 is not dependent on either of the two Hb binding sites contained in the deleted sequences (Figure 1B). However, shortly after the emergence of stripe 2, the maturation process takes on distinct developmental trajectories for the three versions of the enhancer. In particular, over the 18-minute timespan of stripe maturation at 25°C, relative activation of stripe 2 steadily increases in WT, decreases in MSE, and remains nearly constant in INV_MSE. MSE is defective, therefore, in its ability to activate WT levels of stripe 2 gene expression. The dependence of the stripe 2 enhancer on the Hb binding sites for dynamic activation is consistent with the upregulation of Hb expression during cycle 13 and early cycle 14 [36].

Another defect in stripe formation dynamics can be seen with respect to MSE's ability to repress expression in creating the anterior border of stripe 2. In particular, whereas in WT the anterior border grows steadily deeper through the maturation process, evidenced by increasing relative repression (Figure 5F), in MSE the anterior border is overrepressed initially and there is no change over time. A third pattern is seen for the INV_MSE, which exhibits a deepening anterior border, like WT, but at a lower rate. The net consequence of slow INV_MSE peak height and anterior border growth is a visibly derepressed anterior stripe border (Figure 4).

#### Variability in the initial appearance of stripe 2

The sequences removed in creating the MSE transgene include two footprinted Kr binding sites, kr-2 and kr-1. The Kr repressor is expressed in a broad domain in the middle of the early embryo, its anterior boundary of expression establishing the posterior border of Eve stripe 2 [30], [54]. Based on the results presented so far, kr-2 and kr-1 binding sites are neither essential to S2E function, nor are they required for the placement of the Eve posterior stripe 2 boundary, which doesn't differ from WT. Examination of the temporal dynamics of stripe formation, however, identifies a large difference between MSE and WT in the early formation of stripe boundaries (Figure 5F).

**Figure 7:**
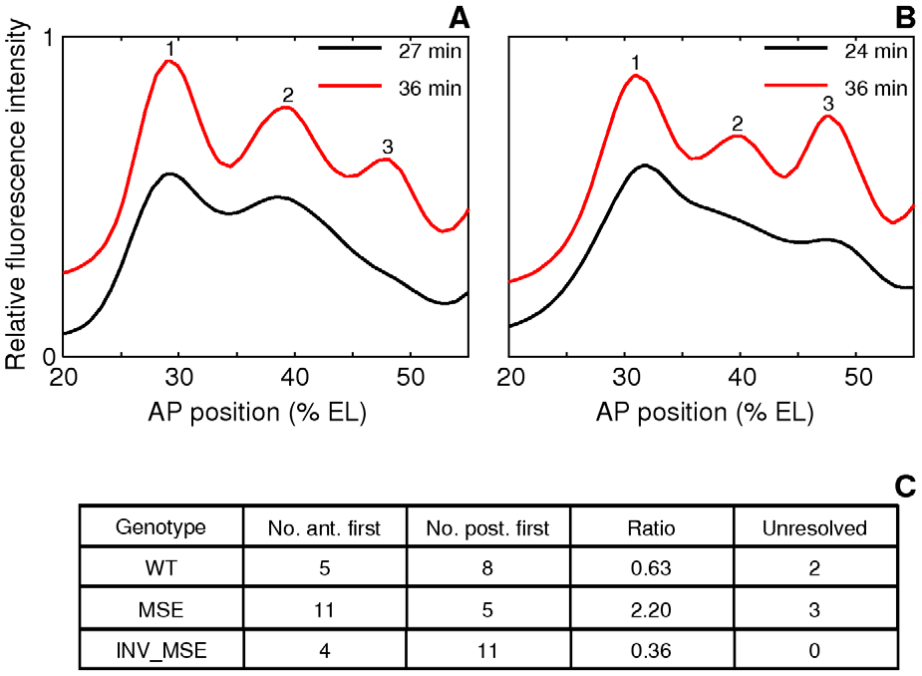
Variation in the order of the appearance of the anterior and posterior borders of stripe 2. A,B. The spline approximations of Eve-YFP expression in two WT embryos showing different order of border formation. The profiles at the later timepoint have been shifted up on the y-axis for clarity. A. An anterior-first embryo. The profile at 27 min has a local minimum at the anterior but not at the posterior, showing that the anterior border formed first. B. A posterior first embryo. The profile at 24 min has a local minimum at the posterior but not the anterior. C. The distribution of embryos in the anterior-first or posterior-first classes. The proportion of anterior-first embryos is much greater in MSE. The last column is the number of embryos that could not be classified because of rapid appearance of both borders within 3 min or noise.

To investigate the early steps in stripe emergence, we used our temporally resolved data in individual embryos to ask the following question: As the early gradient-like expression pattern of Eve resolves into a discernable nascent stripe, does either the anterior or the posterior border of stripe 2 always form first (Figure 7A, 7B)? In 15 WT embryos examined (Figure 7C), we found that this trait varied from embryo to embryo: the anterior border formed first in five embryos, while the posterior border formed first in eight embryos (two embryos could not be resolved). These data confirm, *in vivo*, a result from an analysis of Eve expression in fixed tissue that the order and manner of stripe formation is variable during early cycle 14 [36]. Since gap gene expression levels have large embryo-to-embryo variability at this stage, variation in the order of initiation of Eve border formation may be a direct readout of these fluctuations.

The anterior and posterior borders form by repression by Gt and Kr respectively [30], [31], [32], [54] and we further hypothesized that embryo-to-embryo variation in the expression levels of these two repressors causes the variable temporal order of stripe 2 border creation. If true, we can predict that if kr-2 and kr-1 are functional, their absence in MSE would make this enhancer less sensitive to the repressive effects of Kr, and as a consequence it would not exhibit as strong a bias as WT towards forming the posterior border first. Consistent with this prediction, we observed a ∼2∶1 anterior border-first bias in MSE (Figure 7C) compared to ∼2∶3 ratio in WT. We conclude from these observations that the variation in the manner of stripe 2 emergence is driven by embryo-to-embryo variation in gap gene expression levels, and that the readout of this variation is sensitive to the presence or absence of the sequences missing in the MSE. INV_MSE shows a greater posterior-first bias than WT, foreshadowing the later derepression of its anterior border.

#### Precision of stripe placement

**Figure 8:**
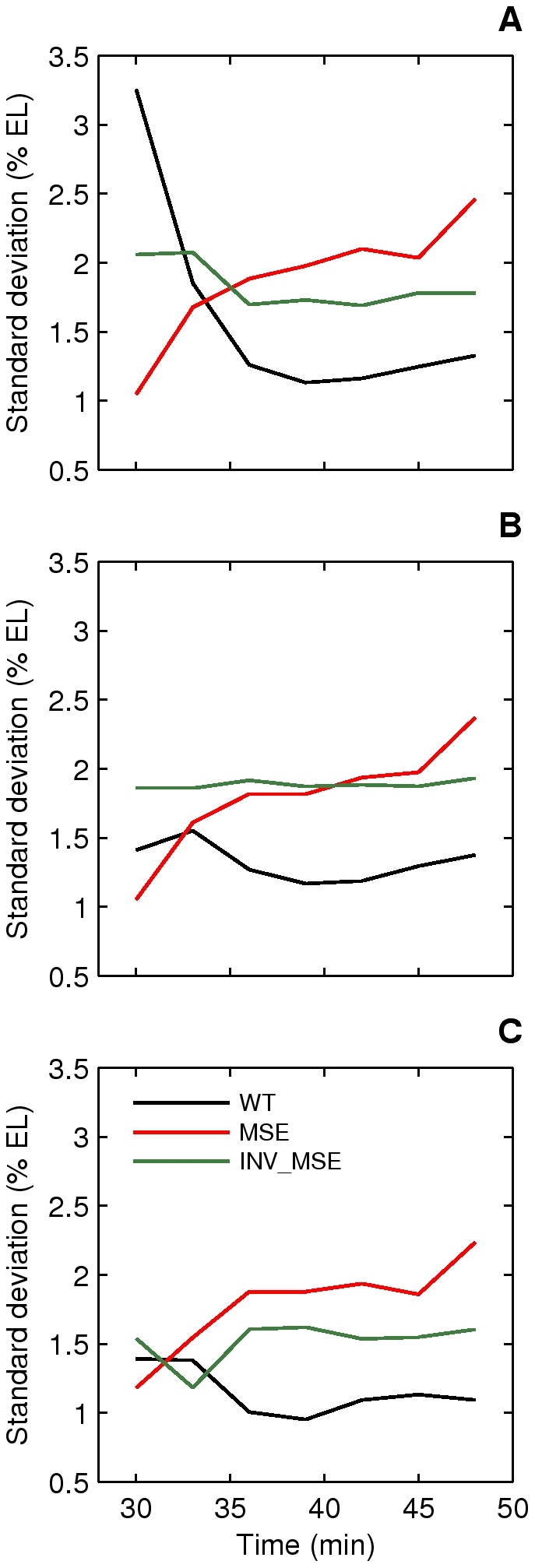
Dynamics of stripe position variation. Standard deviations of peak (A), anterior border (B), and posterior border (C) positions of stripe 2 are plotted as a function of time. The variation in WT stripe 2 positions either decreases (A,C) or remains constant (B) with time and reaches a value between 1–1.5% EL prior to gastrulation. The variation in MSE increases over time to reach a value greater than 2% EL prior to gastrulation. INV_MSE variation is intermediate. N is between 12–15, 14–19, and 9–15 for WT, MSE, and INV_MSE respectively.

Variation in the initiation of the anterior and posterior borders of Eve stripe 2 in WT is temporally resolved to create stereotypical stripe 2 borders. Temporal reduction in positional error is, in fact, a general characteristic of gap and pair-rule expression [36], [56]. In this section we ask whether positional noise in Eve stripe 2 characteristics differ between WT and the MSE. To address this question, we plotted the standard deviations (SD) of estimated locations of the anterior, posterior, and peak positions of stripe 2 as a function of time (Figure 8). Plotting the SD rather than relative positional error is appropriate, as the positions of the peak and borders do not differ between WT, MSE and INV_MSE (Figure S10). As expected, positional variation either remained constant (position of 2A) or decreased over time (position of 2P and peak position), consistent with previous observations [36]. In contrast, all three positional errors increased over time in MSE, leading to a stripe 2 that becomes positionally more variable as it matures. The three traits remained relatively constant for INV_MSE. Since the experimental error arising from sources such as embryo orientation, variable excitation, and background fluorescence is constant in time, any time-dependent effect on positional variance must originate in the intrinsic biological variation of stripe 2 expression. We interpret these results as indicating that sequences contained in the regions deleted in the MSE are required for reducing the variance in Eve stripe 2 positional expression. The other stripe 2 maturation phenotypes, relative activation and repression, did not differ in their variances (Figure S11).

#### Response to temperature perturbation

As a final experiment, we investigated stripe 2 formation in embryos raised at two developmental temperatures, 25°C and 29°C, focusing on the WT and MSE genotypes. The rationale for the experiment is that the two temperatures will create heterochrony in the rates of development. For example, the impact on the diffusion rates of transcription factors is expected to be much smaller than the impact on enzymatic processes [57], including the ones involved in setting the nuclear division rate. Indeed, the length scale of the Bcd gradient, determined by both transport and enzymatic processes, is strongly affected by temperature [34], and nuclear divisions occur much more rapidly at higher temperatures [45]. The question we address here is: Do the WT and MSE enhancers respond differently to developmental temperature perturbation?

Development is considerably more rapid at 29°C. The mean time at which the cell membrane reaches the basal end of nuclei (Figure S12) is 31 min. compared to 38 min at 25°C, and gastrulation begins around 40 *versus* 48 minutes.

**Figure 9:**
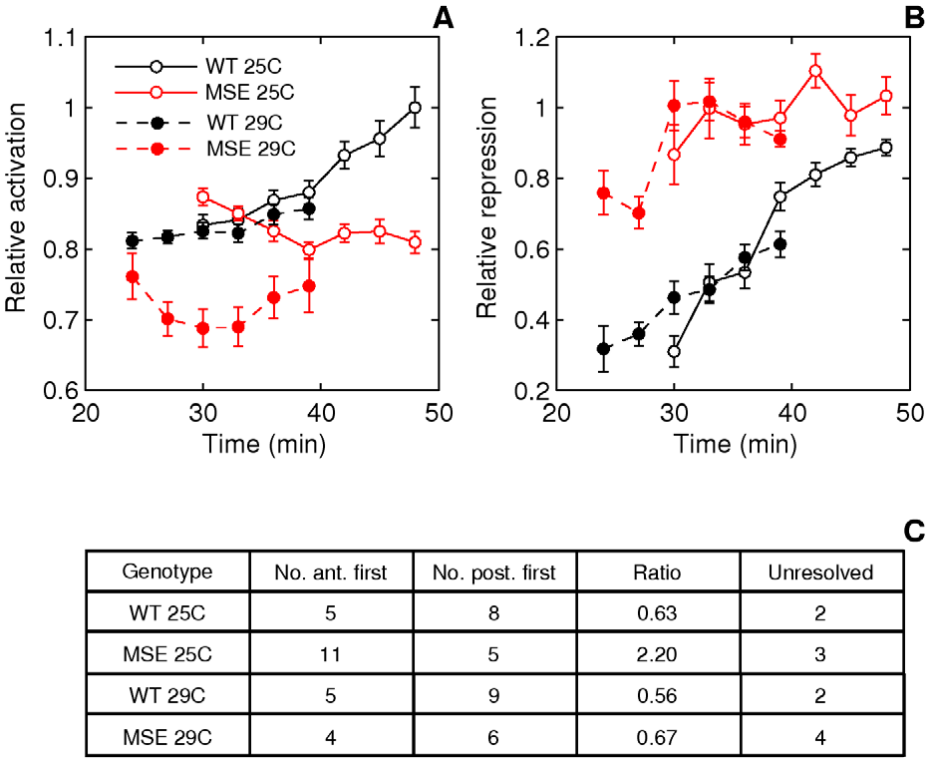
Effect of temperature on stripe 2 maturation and initiation. A. Time series of relative activation of stripe 2. The measurements and plots are as in Figure 5E, 5F. At 29C, WT stripe activation appears to follow the same trajectory as at 25C, except for a time lag. For WT, between 25C and 29C, *p = 0.8054, 0.5617, 0.7238, 0.8516* for *t = 30–39* min. MSE stripe activation at 29C, however, differs both qualitatively and quantitatively from the trajectory at 25C. MSE relative activation is either decreasing or constant, while at 29C it first decreases and then increases. For MSE, between 25C and 29C, *p = 0.0022, 0.0032, 0.0845, 0.3615* for *t = 30–39* min. These phenotypes do not depend on the stripe chosen for normalization (Figure S14B, S14D). B. Time series of relative repression. At 29C, both WT and MSE exhibit trajectories that are similar to, but lagging behind, the ones at 25C. For WT, between 25C and 29C, *p = 0.1798, 0.9445, 0.7238, 0.2201* for *t = 30–39* min. For MSE, between 25C and 29C, *p = 0.2603, 0.6081, 0.6033, 0.8235* for *t = 30–39* min. C. The distribution of embryos forming either the anterior or posterior border first. At 29C, the WT distribution is unperturbed from the distribution at 25C. The MSE distribution at 29C differs from the one at 25C, with a much lower proportion of anterior-first embryos.

The results of a previous study suggest that the *eve* expression pattern scales with developmental time [45], by which we mean that when developmental markers are used to determine time, the Eve expression pattern is qualitatively unchanged upon the application of temperature perturbation. Since we can measure the dynamics of Eve expression quantitatively in absolute time, our data allow us to test temporal scaling. Our data support temporal scaling at a qualitative level, however we found quantitative differences suggesting that the WT pattern at 29°C lags behind the one at 25°C (Figure 9A, 9B). This lag could be due to a differential effect of temperature on YFP maturation *versus* embryonic development and needs further investigation. Our conclusions below do not depend on a particular assumption about temporal scaling (Figure S13).

We assayed the same phenotypes described in the previous section. An emergent stripe 2 is detectable much earlier in MSE at 29°C than at 25°C (Figure 6, *p = 0.0022*), whereas WT stripe initiation times are unchanged (*p = 0.2392*). Similarly, the manner of stripe 2 appearance is perturbed in MSE but not WT. WT maintained its ∼2∶3 posterior-first bias, while MSE switched from a ∼2∶1 anterior-first bias to a ∼2∶3 posterior first bias at 29°C (Figure 9C).

Next, we compare the stripe 2 maturation phenotypes. Given the quicker rate of development at 29°C, we were able to directly compare relative activation and repression time-series (Figure 9A, 9B) at four overlapping time-points. With expression driven by WT, the temporal progression of stripe 2 activation (Figure 9A) is very similar to the one at 25°C. Normalized stripe 2 expression is indistinguishable at the overlapping time-points (*p>0.5*). MSE, however, drives a pattern of activation at 29°C that is both qualitatively and quantitatively different from the one at 25°C. There is a 14-23% reduction in expression level with statistical significance being achieved at 30 and 33 min (*p<0.005*).

The relative repression of the borders of the stripe 2 behaves rather differently from activation. Neither WT nor MSE appear to be perturbed at 29°C (Figure 9B, *p>0.1798*). Similar to its behavior at 25°C, MSE is over-repressed at 29°C.

## Discussion

Eukaryotic enhancers are often structurally compact, characterized by the presence of multiple tightly spaced transcription factor binding sites. Apparently redundant binding sites are present not just within the bounds of empirically-defined minimal enhancers, but also in surrounding genomic regions that are not typically included in reporter assays. However, the redundancy of such binding sites, that is, the sufficiency of the minimal enhancer for biological function, has not yet been firmly established.

In a rigorous experimental test to determine whether an empirically defined eukaryotic minimal enhancer —the eve stripe 2 MSE— is biologically active, we found that it could indeed direct gene expression to engage a normal progression of pattern formation, and produce a fully viable fly when homozygous. The MSE is thus biologically sufficient; it is also essential because when deleted (in *eve^ΔMSE^*), only a rudimentary stripe 2 is formed, *en* stripe 4 is not produced, and embryonic lethality results. Our experiments establish, therefore, that a minimal enhancer is both necessary and sufficient for biological activity.

This conclusion notwithstanding, MSE and INV_MSE are not fully functional because a single copy of either minimal enhancer transgene in hemizygotes strongly reduced embryonic viability (compared to WT) and produced measurable En patterning defects which could be traced to specific patterning defects in Eve stripe 2 expression. This led us to challenge the enhancer with different genetic backgrounds and temperatures, uncovering a role for the peripheral binding sites in directing robust gene expression.

Our experiment with developmental temperature perturbation shows that MSE stripe establishment and maturation dynamics are temperature sensitive. The WT enhancer, in contrast, responds stereotypically to a range of environmental temperatures (25°C and 29°C are both within the range of temperatures experienced by *D. melanogaster* embryos [58]). The complete sequence in WT, besides providing compensation to temperature, is also necessary for the precise placement stripe 2 borders.

The sensitivity of MSE to genetic, environmental, and intrinsic sources of variation expressed at multiple levels of development implies that the structure and composition of the WT enhancer are optimized for robust performance. Wildtype enhancer structure therefore, including the peripheral binding sites investigated here, must have an important functional role among natural populations, which are subject to a large amount of genetic and environmental variability.

The temporal reduction of individual-to-individual variation [36], [42] was the first molecular evidence of developmental canalization [59], [60]. Earlier work has identified specific regulatory interactions, occurring *in trans*, that lead to the canalization of gap gene expression [56], [61]. Here we find that developmental canalization of *eve* stripe 2 expression is also baked into the architecture of the enhancer itself. Due to the divergent effect of gap gene variation on stripe 2 emergence in MSE, we hypothesize that enhancers have evolved the ability to integrate across variability in the upstream signals to which they respond to assure a stereotypical output.

Under this hypothesis, natural selection is expected to continuously tinker with its structural organization to assure that the enhancer functions across the natural range of input factor variability —stochastic, genetic, and environmental. Hence we propose that adaptive fine-tuning of enhancers to perform robustly may be a force driving binding site turnover, a process recently shown to be influenced by positive selection in *Drosophila* enhancers [25]. Such a process might explain why the S2E can experience rapid sequence divergence between closely related species, and yet also exhibit functional convergence across much larger evolutionary timescales [26], [62].

Transcriptional networks employ several distinct tactics to ensure reliable outcomes. Some of these act *in trans*, such as feedback [61], [63] and microRNAs [64], [65], [66], [67], while others, including the role of CRE architecture demonstrated here, act *in cis*: polymerase stalling [68], redundant enhancers [69], [70], [71], and nucleosome depletion [72]. The multiplicity of mechanisms that contribute to robustness but are otherwise dispensable may have evolved to buffer specific perturbations rather than to offer redundancy. Enhancer structure, for example, might filter extrinsic variation in the concentrations of upstream TFs whereas having redundant enhancers is a strategy to reduce the intrinsic noise of transcription by boosting transcription rate. However, it is also possible that the effect of these mechanisms on core function is not detectable by present methods but is sufficient to provide selective advantage at the population level. Core function and robustness would not be entirely separable if that is the case.

Our study was designed to investigate whether the structural features of an eukaryotic enhancer might contribute directly to the robustness of gene expression, as discussed above, but in the course of the study we also found two indications of developmental canalization acting in subsequent stages of development. One of them is the loss of viability in MSE (or INV_MSE) hemizygotes that is largely masked in homozygotes, which we interpret as a manifestation of the segmentation system's capacity to buffer certain stripe 2 defects. Developmental canalization acting downstream of *eve* may also explain the buffering of a sex-dependent difference in Eve stripe 2 expression that we also discovered in the course of this study [73]. Contrary to the general belief that segmentation is sex-independent, Eve stripe 2 expression is in fact sex-specific during cycle 14 in both WT and *eve^+^* embryos [73]. MSE hemizygotes have a strong sex-specific effect on viability (and En patterning) not seen in WT, which implies that sequences contained in WT, but eliminated in MSE, are required for the eventual symmetric segmentation between males and females. Further analysis of the sex-specific Eve stripe 2 phenotype implicated the dosage of *gt*, which being X-linked is present in one copy in males and two copies in females. Lott *et al.* [74] were the first to investigate whether zygotic expression of genes in the early blastoderm showed evidence of dosage compensation prior to the establishment of the conventional dosage compensation mechanism, and discovered that some genes, including *gt*, appeared to be compensated. We were able to demonstrate using genetic analysis that the sex-dependent Eve stripe 2 phenotype is dependent on *gt* dosage [73], implying that the dosage compensation of *gt* must be incomplete. That a difference in Eve stripe 2 expression in males and females is corrected at a subsequent step in development can be taken as additional evidence for the segmentation system's ability to canalize variation in expression arising earlier in development, in this case originating from sex-chromosome linkage of *gt*.

Some, but not all stripe 2 phenotypes in MSE and INV_MSE could be rationalized by the elimination of known Hb and Kr binding sites, including the reduction of peak expression (Hb loss) and the delayed initiation of the posterior border (Kr loss) in MSE. Other phenotypes cannot be readily understood, and considering the differences between stripe 2 formation in MSE and INV_MSE, may be dependent on the specific interaction with factors in neighboring sequences. Evolutionary geneticists have long wondered whether the abundance of large chromosomal inversions between species would be a harbinger for a similar abundance of undetected micro-inversions. Enhancers, after all, are supposed to function with orientation independence. Genome sequence comparison among *Drosophila* species now shows that this is not the case, as the synteny of functional elements within noncoding regions is, in fact, strongly conserved [62], [75]. Our exploration of phenotypic differences between MSE and INV_MSE may provide functional explanation for why noncoding synteny is an evolutionarily conserved trait.

We now return to the question of the physical definition of a *cis-*regulatory element: Do they have sharply defined boundaries? We think not despite the fact that our experiments fall short of a proof. If enhancers were truly discrete, one would imagine that activator occupancy would change discontinuously at the margins. Since there are measurable effects upon the removal of peripheral sequences, we imagine instead that occupancy decays continuously as one moves away from the empirically defined “core”. Due to the promiscuity of transcription factor binding, this occupancy distribution might extend well beyond the conventional boundaries of the core enhancer and will change with tissue and time. Environmental and genetic perturbation will also change the relative weights of the core and periphery. The evolutionary process of binding site turnover, in turn, ensures that enhancers maintain stereotypical output across a wide range of conditions.

## Materials and Methods

### Relative viability

Each transgenic rescue line was crossed into the *eve^R13^* (R13) mutant background, generating flies of the genotype *w; b*,R13 */CyO;* attP2[*S2E^A1^EVE^A2^*]*/TM3,Sb*. A1 refers to the wildtype (wt), MSE or INV_MSE alleles of the stripe 2 enhancer (S2E) and A2 to two alleles of *eve* coding region, native or tagged with YFP. F1 adults were scored for 2^nd^ and 3^rd^ chromosome phenotypic markers (*Cy, b,* and *Sb*), to identify the relevant transgene rescue genotypes (Figure 2A). Replicate crosses were established between 10 females and 20 males in two large culture vials at 25°C. Parents were transferred to a fresh culture vial every day for 20 days. The emerging adult offspring were collected every day from the culture vials for a period of 10 days for scoring. This approach ensured that mutants with slow development rates were counted.

Relative viability of flies carrying one or two copies of the *eve* transgene and lacking a functional endogenous *eve* gene was determined from the number of adult survivors. Expected viability for these genotypes was calculated based on the count of flies carrying one copy of the endogenous *eve* locus and either one or two copies of the transgene-bearing third chromosome (Figure 2A, *R* *13/CyO;* attP2[*S2E^A1^EVE^A2^*]*/TM3,Sb* and *R13/CyO;* attP2[*S2E^A1^EVE^A2^*], respectively). Employing this procedure, we estimated the relative viability of four different transgene genotypes: 16.4 kb *eve* without (EVE) and with (WT) the YFP fusion and 16.4 kb *eve* with YFP fusion carrying either the MSE (MSE) or INV_MSE (INV_MSE) enhancer. In all four cases, the observed ratios of the two genotypes carrying one copy of the endogenous *eve* locus and either one or two copies of a transgene conformed to the expected 2∶1 segregation ratio (Tables S1 and S2), implying wildtype viabilities for both. On this basis, the numbers of adults produced by these two genotypes were combined to calculate the expected numbers for the rescue transgene genotypes. The comparison of relative viabilities of WT, MSE and INV_MSE versions of the transgene is predicated on the assumption, therefore, that the two genotypes carrying one copy of the native *eve* locus and one or two copies of the specific transgene are fully viable, a reasonable assumption given that all three stripe 2 enhancer transgenes alone rescued flies to adulthood when homozygous (as described in the Results).

To evaluate the ability of the transgenes to rescue R13, *Df(2R)eve* (*Df(eve)*), or *eve^ΔMSE^* lethality when present in one copy, we crossed eight healthy females of the genotype *w; b*,R13 */CyO*, or *w; Df(eve)/CyO*, or *w; eve^ΔMSE^/CyO*, with 15 healthy males of the genotype *w; b*,R13; attP2[*S2E^A1^EVE^A2^*] in large vials at 25°C. Scoring of emerging adult offspring was as described above.

### Constructs

WT, MSE and INV_MSE constructs (Figure 1B; sequences in Text S1) were created by recombineering with Red/ET counter-selection BAC Modification Kit (Gene Bridge http://www.genebridges.com/gb/pdf/K002_Counter_Selection_Kit-V3.0-2007.pdf). The BAC R06J01 was used as the DNA source for the genomic *eve* locus.

*eve*-containing DNA, from -6.6 kb to +9.8 (Figure 1A), was cloned into attB_3xP3_DsRed_P15A-Amp by recombineering (vector sequence and primers are provided in Text S1). This *eve*-containing DNA fragment is slightly larger then the fragments EGN84 (−6.4 kb to +8.4 kb), EGN86 (−6.4 kb to +8.6 kb), and EGN92 (−6.4 kb to +9.2 kb) previously used in rescue assays [47], [50]. The rescue potencies of EGN84, EGN86, and EGN92 have varied, depending on place of transgene insertion in genome, but never exceeding 40% homozygous rescue. The choice of our fragment, -6.6 kb to +9.8, was motivated by the desire to maximize the span of *eve*-containing DNA bounded by the neighboring genes CG12134 and TER94 and to include experimentally identified endogenous insulator regions (http://www.modencode.org/). This was possible because the recombineering method does not require restriction sites.

The *eve-YFP* fusion construct was also created by recombineering (primers and sequences are provided in the Text S1). The SYFP2 superfolder, a rapidly maturing version of YFP (generously provided by Ben Glick, University of Chicago), was added to the C-terminus of the *eve* peptide. The half-life of maturation of the SYFP2 in yeast is ∼8–10 min at 30C (Ben Glick, personal communication).

### Structure of altered S2E enhancers

The S2E (798bp) is bordered on the 3′ and 5′ sides by completely conserved blocks of 18bp and 26bp, respectively (marked as blocks **a** and **b** in Figure 1B; [27]), which are the generally accepted boundaries of the enhancer. The footprinted binding sites for upstream transcription factors have been described [31], [76]. To create the MSE we deleted two fragments, 33bp and 211bp long, from the distal and proximal regions of S2E respectively (shown as rectangles in Figure 1B). The classic MSE (Minimal Stripe 2 Element) contains 480bp [32]; our MSE adds an additional 29bp distally to avoid the fusion of two repressor binding sites, kr-6 and kr-3, in the INV_MSE construct. This additional native sequence does not contain any mapped TFBS. The 509bp-long MSE and INV_MSE both lack two Kr binding sites (kr-1 and kr-2) and two Hb binding sites (hb-2 and hb-1) and both are bordered by identical sequences. kr-1 and kr-2 are evolutionarily conserved among all species in the melanogaster subgroup [27]. hb-2 differs by only one change between *D. melanogaster* and *D. picticornis*, indicating that this sequence must be functionally constrained [27]. In contrast, hb-1 is a relatively young binding site that is present in *D. melanogaster* and emerged within the melanogaster subgroup [26], [27]. We considered substituting a random sequence for the deleted sequences, but decided against this course of action to avoid the unintended creation of binding sites for unknown TFs.

### The attB vector for integration

We created a novel docking site integration vector, the 3265 bp-long attB_3xP3_DsRed_P15A-amp (sequence in Text S1). The compact size of the vector facilitated its amplification by PCR, a necessary step in recombineering. The vector contains an attB sequence for integration, a fly transformation marker, DsRed, expressed in the eye by the PAX-6 promoter, and the p15A origin of replication site that aids in cloning large fragments (up to 50 kb).

### Site-specific integration of attB for plasmids into attP2 landing site

Site-specific integration was carried out by co-injection with phiC31-integrase RNA as described [48], [49]. The attP2 *D. melanogaster* stock was provided by M. Markstein. Integration of the vector attB into the attP2 landing site was verified by using two pairs of primers (see Text S1). The choice of the docking attP2 site was according to [49].

### Drosophila strains

*Df(2R)eve*, *eve^R13^*, and *eve^ΔMSE^*. *Df(2R)(eve)* is a deficiency that includes at least five lethal complementation groups [77], [78]. The R13 is null mutation that truncates the protein within the homeodomain [47]. We created the *eve^ΔMSE^* lethal mutant by replacing the 480bp fragment corresponding to the MSE from the endogenous *eve* locus with the *white^+^* gene using ends-out homologous recombination according to the methods described in [79], [80], [81]. DNA fragments homologous to approximately 4 kb and 3.5 kb of the *eve* sequences flanking the MSE were cloned into the pTV2 vector. The donor DNA construct was transformed into the germline of *Drosophila melanogaster* by P-element-mediated germ line transformation [82].

All three lethal mutations were balanced over the marked balancer chromosome *CyO, p[hb-lacZ]* to allow the identification of mutant embryos by immunostaining for β-galactosidase or by PCR analysis for the *β-galactosidase* gene. PCR-based genotyping of individual Drosophila embryos after immunostaining is described in [26]. Also, the R13 null mutant was balanced over the fluorescently marked balancer chromosome *CyO,p[Dfd-YFP]* to allow the identification of mutant larvae.

### Analysis of embryos in fixed tissue

Embryo collection and fixation was as described [83]. *Drosophila* embryos were immunostained with Rabbit polyclonal anti-Eve (provided by Mark Biggin, University of California, Berkeley, 1∶1,000 dilution) and Chicken polyclonal anti-GFP (abcam, USA, 1∶3,000 dilution) primaries and Alexa Fluor-546, Alexa Fluor-647 Goat anti-Rabbit IgG (Molecular Probes, Invitrogen, 1∶400 dilution), and Alexa Fluor-647 goat anti chicken IgG (Molecular Probes, Invitrogen, 1∶400 dilution) secondaries.

Histochemical staining with anti-En monoclonal 4D9 at 1∶10 dilution was visualized using HRP-DAB enhanced by nickel [83].

*in situ* hybridization was carried out as described [27], [42].

### Live imaging

We recombined the attP2[*S2E^A1^EVE^YFP^*] and P[His2Av-mRFP1] (Bloomington stock 23650) transgenes onto the same third chromosome to visualize Eve-YFP and nuclei in parallel. Due to the low fertility of the recombinant homozygous genotype, we imaged embryos from a cross between attP2[*S2E^A1^EVE^YFP^*] males and attP2[*S2E^A1^EVE^YFP^*],P[His2Av-mRFP1]/TM3,Sb females. The His-RFP fluorescence observed in cleavage cycle 14 is primarily maternal since mRFP1 matures slowly. As a result, we imaged individual embryos carrying either one or two copies of Eve-YFP. We could make comparisons between lines and treatments because we always normalized stripe 2 expression levels to that of stripe 1 (Figure 4B) or the anterior border height to that of the posterior border (Figure 5A).

We maintained flies at a constant temperature, either 25C or 29C, and collected embryos on apple juice plates for 1–1.5 hours. The embryos were allowed to age for another hour before being dechorionated in 50% bleach for 3.5 min. The embryos were then mounted on circular coverslips for a temperature control chamber (FCS3, Bioptechs Inc., Butler, PA, USA). We oriented the embryos laterally by hand and placed a drop of 700 series Halocarbon oil such that embryos at the edge of the drop were partially exposed to air. The chamber was mounted on the microscope stage and heated to the target temperature. The objective was also heated to ensure a uniform and stable temperature distribution. The period of time for which the embryos were at room temperature, during dechorionation and mounting, was not more than 15 min.

We observed a significant attenuation of YFP signal in embryos completely covered by Halocarbon oil, although their development appeared normal. This is consistent with the quenching of GFP fluorescence in anaerobic conditions [55]. For this reason we only chose embryos that were partially exposed to air for imaging.

We identified a cycle 13 embryo and observed it in the histone channel, undergoing the thirteenth nuclear division. To allow a comparison of embryos in absolute time, we started the clock during the final mitotic wave [84] from telophase to cycle 14 interphase. This wave is identifiable by the presence of cycle 14 interphase nuclei at the poles and telophase nuclei at the equator of the embryo. On average mitotic waves last 0.5 min [84], allowing us to determine the age of each embryo during imaging within ±0.5 min. After this point, we imaged the embryo at the midsagittal plane every 3 min for an hour, when gastrulation movements begin.

### Confocal microscopy

We imaged embryos using a Vti Infinity 3 multipoint confocal system (Visitech International, Sunderland, UK) mounted on a Zeiss AxioPlan 2 microscope (Carl Zeiss, Inc., USA). A backthinned EMCCD camera (Hamamatsu Photonics UK Ltd, Hertfordshire, UK) having a resolution of 512x512 pixels and 16 bit depth was employed for fluorescence detection.

For YFP, excitation was provided by a 514 nm solid state laser with a triple band (442 nm, 514 nm, 633 nm) dichroic. A dual band (450–490 nm and 515–580 nm) emission filter was used for detection. mRFP1 emission is less than 10% of peak for wavelengths less than 580 nm, minimizing crosstalk. The 561 nm laser line, a dual band dichroic (420 and 560 nm) and 585 nm long pass emission filter were used for detecting mRFP1.

The frequency of image acquisition (every 3 min) and exposure time (2–5 sec) were chosen conservatively to minimize photobleaching and maximize signal to background ratio. At this frequency of acquisition, we observed signal attenuation with time only at an exposure of 10 sec. With a 2–5 sec exposure, the brightest embryos had a few pixels at the maximum detection limit of the camera. All embryos within a set of experiments (either 25C or 29C) were acquired with the same microscope settings.

### Image segmentation

We adapted an algorithm used to segment images of immunofluorescently-stained flattened embryos [85] for use with live image data from the midsagittal plane. The original algorithm works in two steps, 1) a whole embryo mask is used to rotate, orient, and crop the image and 2) after smoothing, the watershed algorithm is applied to separate the nuclei and make a nuclear mask.

We made two modifications that enable fully automated segmentation save for the user input required in orienting the embryo. First, instead of a whole embryo mask we create a cortical mask since yolk autofluorescence leads to spurious detection of nuclei. We use the gray-scale top-hat transformation [86] followed by Otsu thresholding to remove yolk autofluorescence. Next, erosions followed by morphological reconstruction on the thresholded binary image remove the autofluorescent vittelline membrane. Second, we correct oversegmentation caused by the invaginating cell membrane during the middle of cycle 14. We compute the neighbors of each watershed region. A pair of watershed regions is considered to belong to the same nucleus if 1) they are separated by a watershed line greater than one pixel in length and 2) one region touches the yolk but not the outside while the other touches the outside and not the yolk. The members of such pairs are then fused. The mean Eve-YFP fluorescence is calculated in the mask corresponding to each nucleus and is saved along with the coordinates of the centroid for further processing.

### Feature detection

We used the CSAPS function of MATLAB (MathWorks Inc.) to determine a smoothed cubic spline approximation to the fluorescence data. Given data vectors *x* and *y*, CSAPS determines a spline, *f(x)*, that minimizes a cost function that is a sum of the total error made by the spline in approximating the data and a measure of the ‘roughness’ of the spline. The relative weight of these two terms is controlled by the roughness parameter *p*. *p = 1* yields a perfect, but non-smooth, fit, whereas *p = 0* gives a least-squares straight line fit to the data. We chose a value, *p = 0.5*, to give a good fit without detecting spurious extrema and used the same value for all embryos. The mean error of the spline approximation at the extrema was ∼10% at this value of *p*.

The spline was used to estimate the positions and fluorescence levels at the extrema in each embryo (Figure 5B). Border positions were calculated as the position where the fluorescence level is at the mean of the consecutive extrema. The height of a border is the difference in fluorescence levels at consecutive extrema.

In order to determine which border formed first (Figure 7A, 7B), we used the spline to follow the temporal evolution of the Eve pattern (Video S1). In each embryo, we noted the order in which the minima immediately anterior and posterior to the stripe 2 peak were first detected by the spline. We ensured that these minima were genuine by following them through time and confirming that they developed into mature interstripes.

## Supporting Information

Figure S1 Adult viability in the hemizygous rescue of the lethality of the R13 mutant. Example cross and offspring genotypes for the viability assay (see Methods for details). Genetic notation — *CyO* and *TM3* are the second and third chromosome balancers respectively; *b:* mutant allele of *black;* orange box: native *eve;* R13 and X'd out orange box: R13 lethal mutant; attP2: docking site; yellow box: transgene attP2[*S2E^A1^EVE^YFP^*]. A1 indicates the allele of S2E used (wt, MSE, or INV_MSE). The table shows the offspring genotypes (first column), the sex (second column), and the number of eclosed adults counted for WT, MSE, and INV_MSE in the third, fourth, and fifth columns respectively.
(TIF)

Figure S2 Adult viability in the hemizygous rescue of the lethality of the *Df(eve)*/R13 heterozygote. The crosses and table are as in Figure S1.
(TIF)

Figure S3 Adult viability in the hemizygous rescue of the lethality of the *eve^ΔMSE^*/R13 heterozygote. The crosses and table are as in Figure S1.
(TIF)

Figure S4 Viability of first instar larvae in the hemizygous rescue of the lethality of the R13 mutant. The crosses are as in Figure S1 with the exception that the second chromosome balancer has a P-element insertion of *Deformed-YFP* (*Dfd-YFP*) that allowed the scoring of larvae carrying the balancer.
(TIF)

Figure S5 *eve^ΔMSE^* causes embryonic lethality. The cross on top is between balanced *eve^ΔMSE^* lines. The balancer carries a P-element insertion of *Dfd-YFP*. The offspring genotypes are in the first column, the second column indicates whether a genotype is expected to be Dfd-YFP positive or not and the third column has the number of hatched larvae counted. The third genotype is not observable because the *CyO/CyO* homozygote is embryonic lethal.
(TIF)

Figure S6 The lengths of parasegments 3 and 4 in the En pattern were measured at the ventral midline of embryos. Shown by green and yellow bars respectively.
(TIF)

Figure S7 Parasegment 3 is reduced in the hemizygous rescue of *eve^ΔMSE^*/R13 by the MSE or INV_MSE transgenes. A. Schema to study the En pattern in the *eve^ΔMSE^* /R13 mutant embryos rescued by the altered *eve* transgenes. Example cross and relevant offspring genotypes for the assay. Genetic notation is the same as in Figure S1 with the exception that the second chromosome balancer has a P-element insertion of *hb-lacZ* that allowed the scoring of embryos carrying the balancer. The R13/*eve^ΔMSE^* mutant embryos were identified by the absence of β-galactosidase expression and PCR genotyping. B. The En pattern in the R13/*eve^ΔMSE^* mutant embryos with *eve* driven by one copy of the WT, MSE, or INV_MSE transgenes; stage 11. Note the variation in parasegments 3 and 4. C. Difference between WT and MSE (or INV_MSE) in En stripe 4 spatial expression was evaluated as the ratio of the length of parasegment 3 to the sum of parasegments 3+4 (see Methods and Figure S6). Error bars are standard deviations. N for each case is about 20.
(TIF)

Figure S8 Eve-YFP faithfully reproduces endogenous Eve expression. Embryos carrying the Eve-YFP transgene were costained with anti-Eve and anti-GFP antibodies and imaged in a confocal microscope. The images were segmented (see Methods) and mean anti-Eve or anti-GFP fluorescence was calculated in each nucleus. A. A scatter plot of anti-Eve with anti-GFP fluorescence in all nuclei of 10 embryos. The data for each embryo are plotted with the same color. The values for each channel were normalized to maximum fluorescence observed in the embryo, but were not manipulated otherwise. The scatter shows strong proportionality as it lies along the diagonal (*r^2^>0.88* for all embryos). B. anti-Eve and anti-GFP profiles extracted from a dorsoventral strip along the anteroposterior axis of the same embryo. Each profile is normalized to its maximum expression. The anti-GFP profile, mostly overlapping with the other, has lower expression in some stripes, most notably stripes five and six. Stripes 2-7 steadily increase expression during middle cycle 14 to achieve a level of expression equal to that of stripe one [35], with stripes five and six the last to reach maximum expression. The lower expression of the other stripes then suggests that the anti-GFP profile simply lags behind the anti-Eve profile. However, both profiles appear to belong to time class T6 of the staging scheme of Surkova *et al*. [36] implying that the lag is less than 6 min. C. Scatter plot of normalized border heights 2A-7A (see Methods). Border height eliminates the effect of background staining, since it is calculated as the difference in the expression of two closely spaced points in the embryo. Border heights also lie along the diagonal reflecting the proportionality of Eve-YFP expression to that of endogenous Eve. Border heights were extracted from the sample of embryos shown in panel A. N = 10. D. Scatter plot of stripe positions. *r^2^>0.9998*. Different positions are shown in different colors. Stripe positions were extracted from the sample of embryos shown in panel A. N = 10.
(TIF)

Figure S9 MSE and INV_MSE phenotypes in fixed tissue. Confocal images of mid cycle 14 embryos immunostained for YFP. A,D. WT, B,E. MSE, and C,F. INV_MSE. D-F. Magnified view of the anterolateral region. Arrows point to stripe 2. Stripe 2 expression is weaker in MSE and the anterior border is derepressed in INV_MSE, validating the phenotypes observed in live data.
(TIF)

Figure S10 Positions of the peak and borders of stripe 2 do not differ between WT and MSE or INV_MSE. Plots and sample sizes are as in Figure 5. No statistically significant differences were observed (*p>0.09* for all comparisons between WT and MSE or INV_MSE). A. Peak of stripe 2. B. Anterior border. C. Posterior border.
(TIF)

Figure S11 The variation of relative activation and repression of stripe 2 does not differ between WT and MSE or INV_MSE. Plots show time series of the standard deviation of the relative activation (A) and relative repression (B). Sample sizes are as in Figure 5.
(TIF)

Figure S12 Accelerated rate of development at 29C. Using the histone channel of embryo movies (see Methods), we noted the time during cellularization when the cell membrane is at the basal end of nuclei [36], an easily identifiable morphological mark. The histograms of these times are shown for embryos developing at either 25C or 29C. The membrane reaches the basal end of nuclei at 38.2 (±2.2) min at 25C and at 30.6 (±2.5) min at 29C.
(TIF)

Figure S13 The effect of scaling time according to a developmental mark on the relative activation and repression of stripe 2. We plot the 29C data (Figure 9) at timepoints that are the product of absolute time with the ratio of the developmental rate at 29C to the rate at 25C (Figure S12). Our conclusions about the differential effect of temperature on these phenotypes are robust to such scaling. The relative activation of MSE at 29C still follows a progression that is qualitatively and quantitatively different from the one at 25C. However, the interpretation of the effect of temperature on WT differs. In absolute time, the phenotypes appear to follow the same trajectories at 29C as 25C except for a lag whereas, scaled to developmental time, there appears to be a deficit in the relative activation and repression of stripe 2. A. Relative activation. B. Relative repression.
(TIF)

Figure S14 Time series of stripe 2 expression normalized by the expression of stripes 3 or 6. See Figure 5 and Figure 9 for an explanation of plots and sample sizes. A,B. Stripe 2 expression normalized to stripe 3 expression. C,D. Stripe 2 expression normalized to stripe 6 expression. A,C. Time series for 25C. Unlike stripe 1 expression, which is higher than stripe 2 throughout cycle 14, stripes 3 and 6 are lower than stripe 2 initially but increase in expression as the cycle progresses. As a result, stripe 2 expression normalized to these stripes displays a decreasing trend for WT instead of an increasing one (Figure 5E). However, normalized MSE expression is lower than WT and INV_MSE is intermediate, consistent with the phenotypes observed when normalizing with stripe 1. A. Between WT and MSE, *p = 0.0143, 0.0188, 0.0150, 0.0275, 0.004, 0.0097, 0.089*. Between WT and INV_MSE, *p = 0.109, 0.1481, 0.6104, 0.9817, 0.4553, 0.229, 0.3947*. C. Between WT and MSE, *p = 0.0163, 0.0063, 0.001, 0.0009, 0.0007, 0.0008, 0.0062*. Between WT and INV_MSE, *p = 0.4757, 0.0464, 0.0308, 0.0628, 0.0014, 0.0016, 0.0121*. B,D. Comparison of time series between 25C and 29C. As in Figure 9A, the time series at 25C and 29C overlap for WT, but not for MSE. B. For WT, between 25C and 29C, *p = 0.3687, 0.1092, 0.1904, 0.253*. For MSE, *p = 0.0002, 3.1886e-05, 0.0001, 0.0019*. D. For WT, *p = 0.0149, 0.5308, 0.8843, 0.3496*. For MSE, *p = 0.014, 0.0007, 0.0011, 0.001*.
(TIF)

Table S1 Adult survival and relative viability for the EVE transgene.
(DOC)

Table S2 Adult survival and relative viability of the WT, MSE, and INV_MSE transgenes.
(DOC)

Table S3 Ratio (*X*) of En parasegment 3 length relative to 3+4 in hemizygous embryos (*Df(eve)/*R13*).*
(DOC)

Table S4 Ratio (*X*) of En parasegment 3 length relative to 3+4 in hemizygous embryos (*eve^△MSE^* /R13*).*
(DOC)

Text S1 Primers and sequences.
(DOC)

Video S1 Eve-YFP expression in a WT embryo during cleavage cycle 14.His2Av-RFP expression (top), Eve-YFP expression (middle), and mean nuclear Eve-YFP fluorescence intensities from the ventral side (bottom) are shown. Anterior is left and dorsal is above. In the bottom panel, yellow circles are data and the white line is a spline approximating the data. The *x*-axis is the AP position in % EL and the *y*-axis is the normalized fluorescence intensity. Time is measured from the completion of the thirteenth nuclear division (see Methods) and gastrulation movements begin at ∼50 min.
(MOV)
