# Supplementary figures and images for: Consequences of Eukaryotic Enhancer Architecture for Gene Expression Dynamics, Development, and Fitness
Source: PLoS Genet. 2011 Nov 10;7(11):e1002364. doi: 10.1371/journal.pgen.1002364 (PMC3213169; doi:10.1371/journal.pgen.1002364)

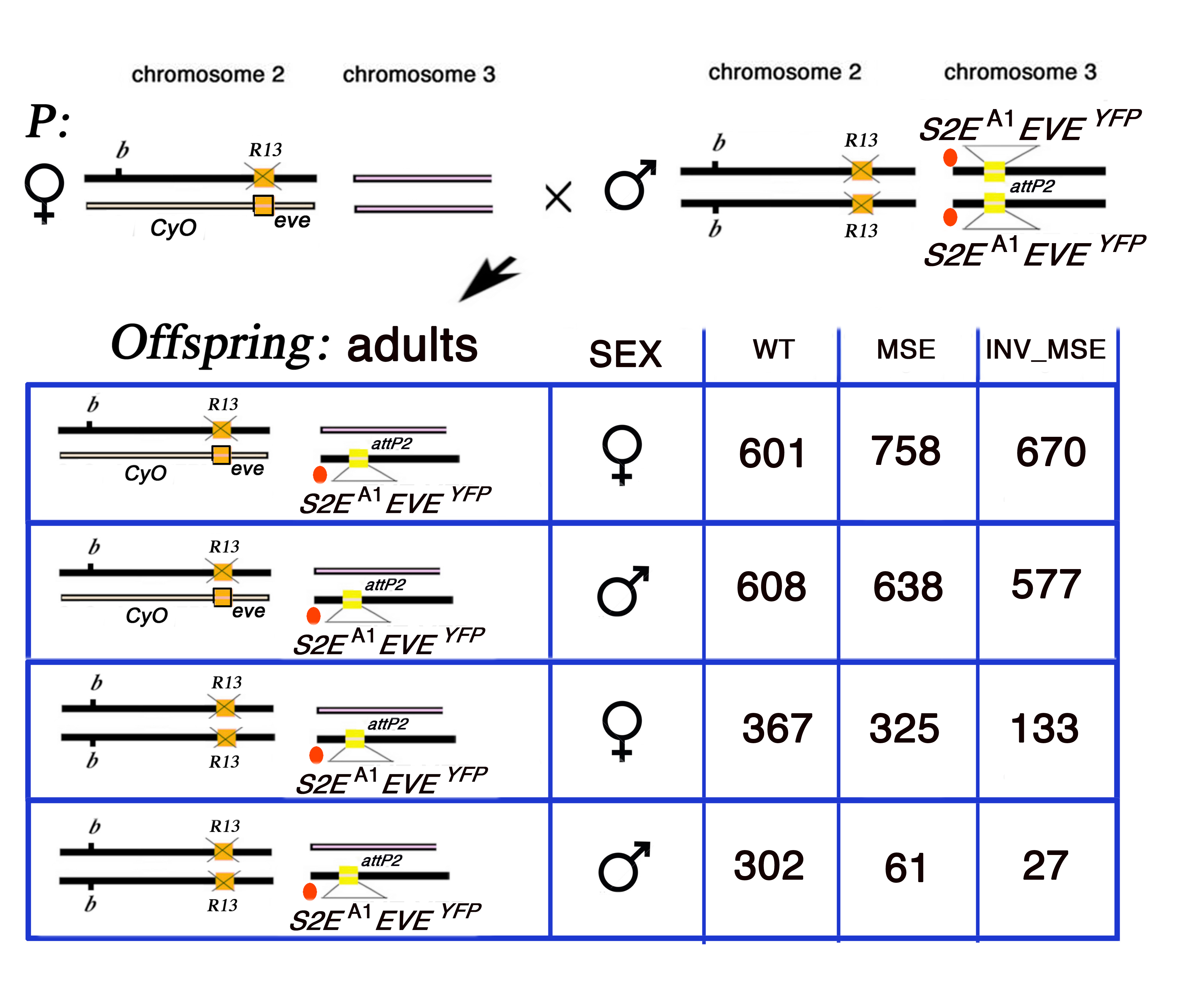

Supplement: Figure S1 — Adult viability in the hemizygous rescue of the lethality of the R13 mutant. Example cross and offspring genotypes for the viability assay (see Methods for details). Genetic notation — CyO and TM3 are the second and third chromosome balancers respectively; b: mutant allele of black; orange box: native eve; R13 and X'd out orange box: R13 lethal mutant; attP2: docking site; yellow box: transgene attP2[S2EA1EVEYFP]. A1 indicates the allele of S2E used (wt, MSE, or INV_MSE). The table shows the offspring genotypes (first column), the sex (second column), and the number of eclosed adults counted for WT, MSE, and INV_MSE in the third, fourth, and fifth columns respectively. (TIF) [file pgen.1002364.s001.tif]

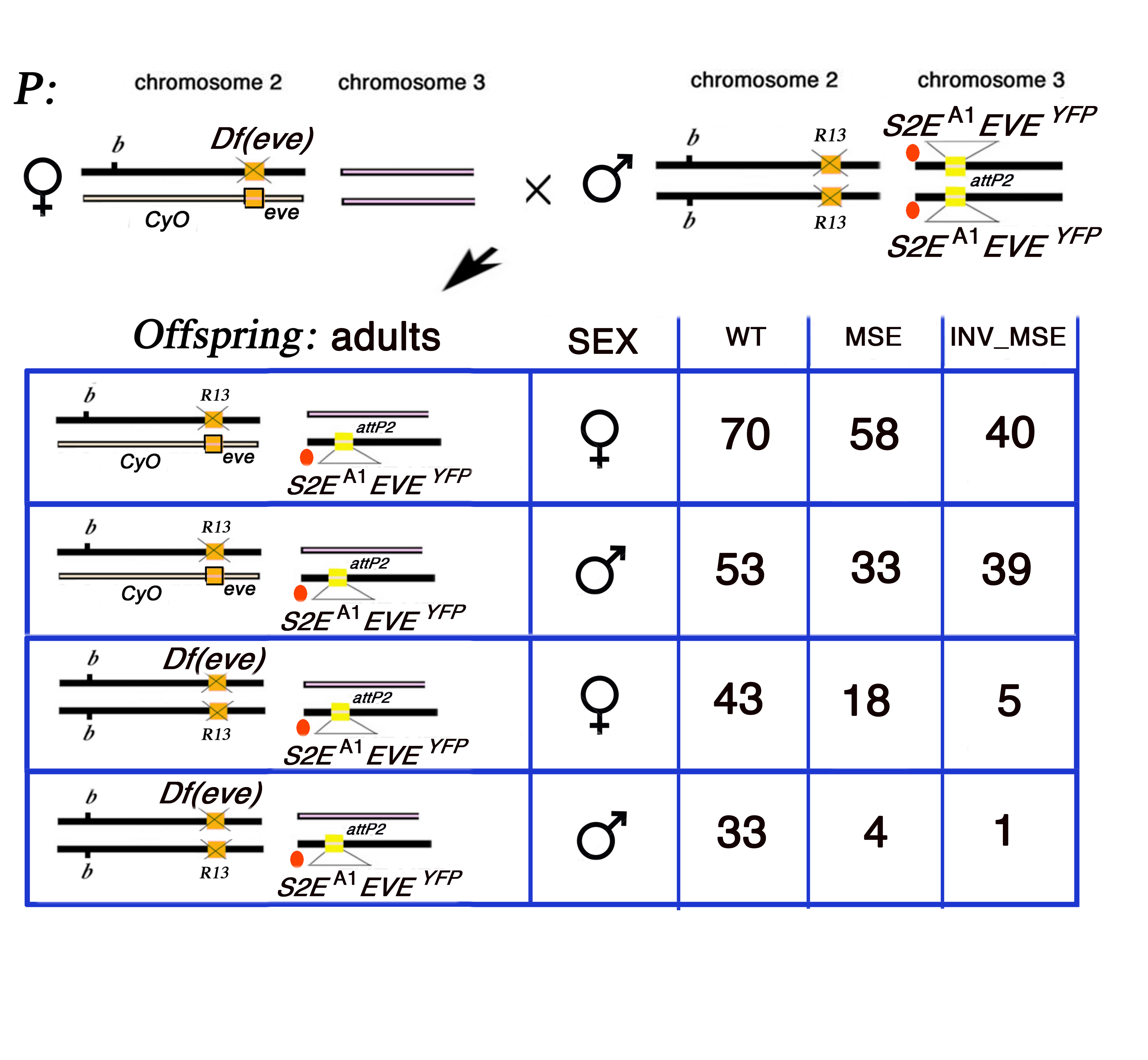

Supplement: Figure S2 — Adult viability in the hemizygous rescue of the lethality of the Df(eve)/R13 heterozygote. The crosses and table are as in Figure S1. (TIF) [file pgen.1002364.s002.tif]

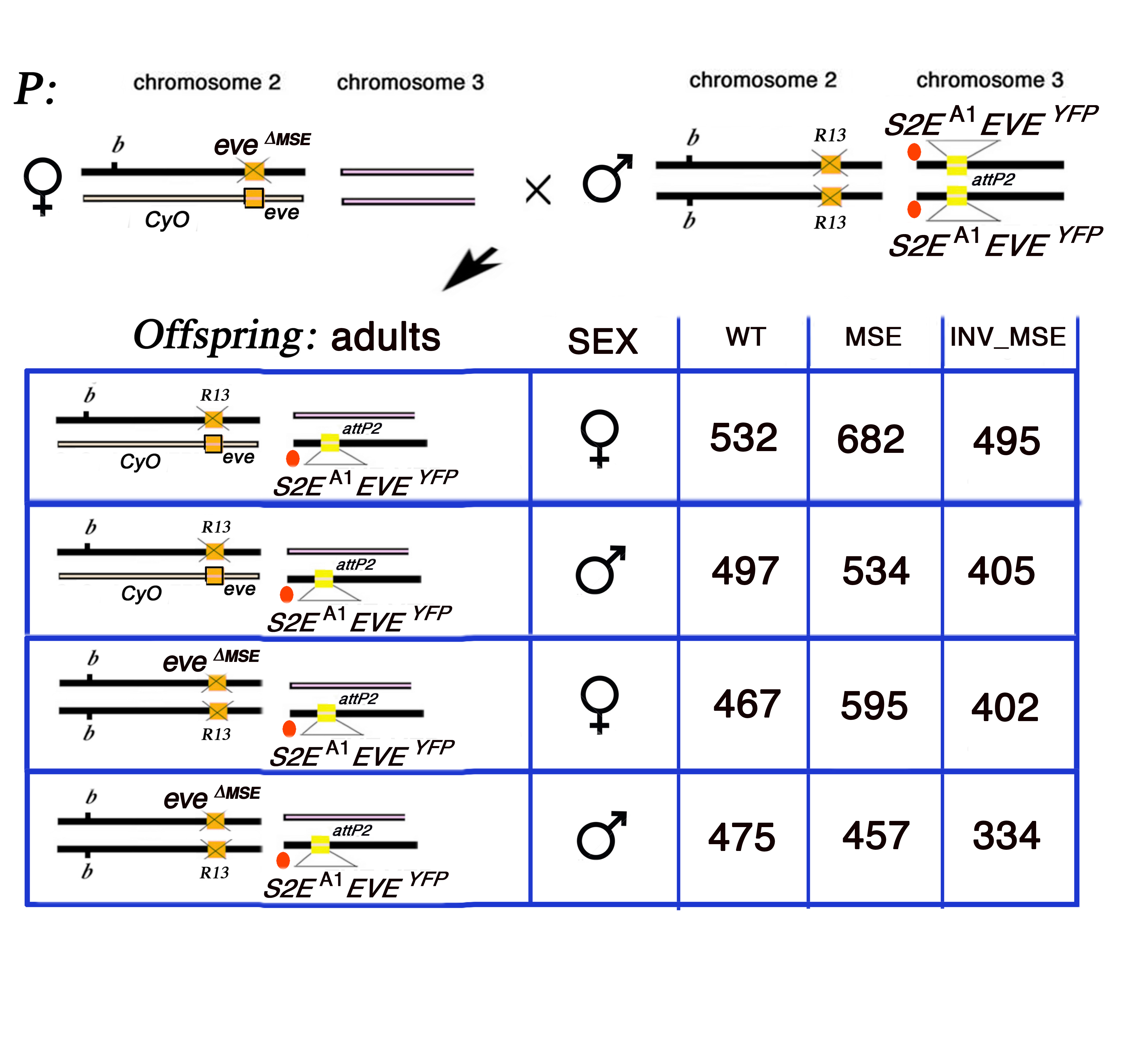

Supplement: Figure S3 — Adult viability in the hemizygous rescue of the lethality of the eveΔMSE/R13 heterozygote. The crosses and table are as in Figure S1. (TIF) [file pgen.1002364.s003.tif]

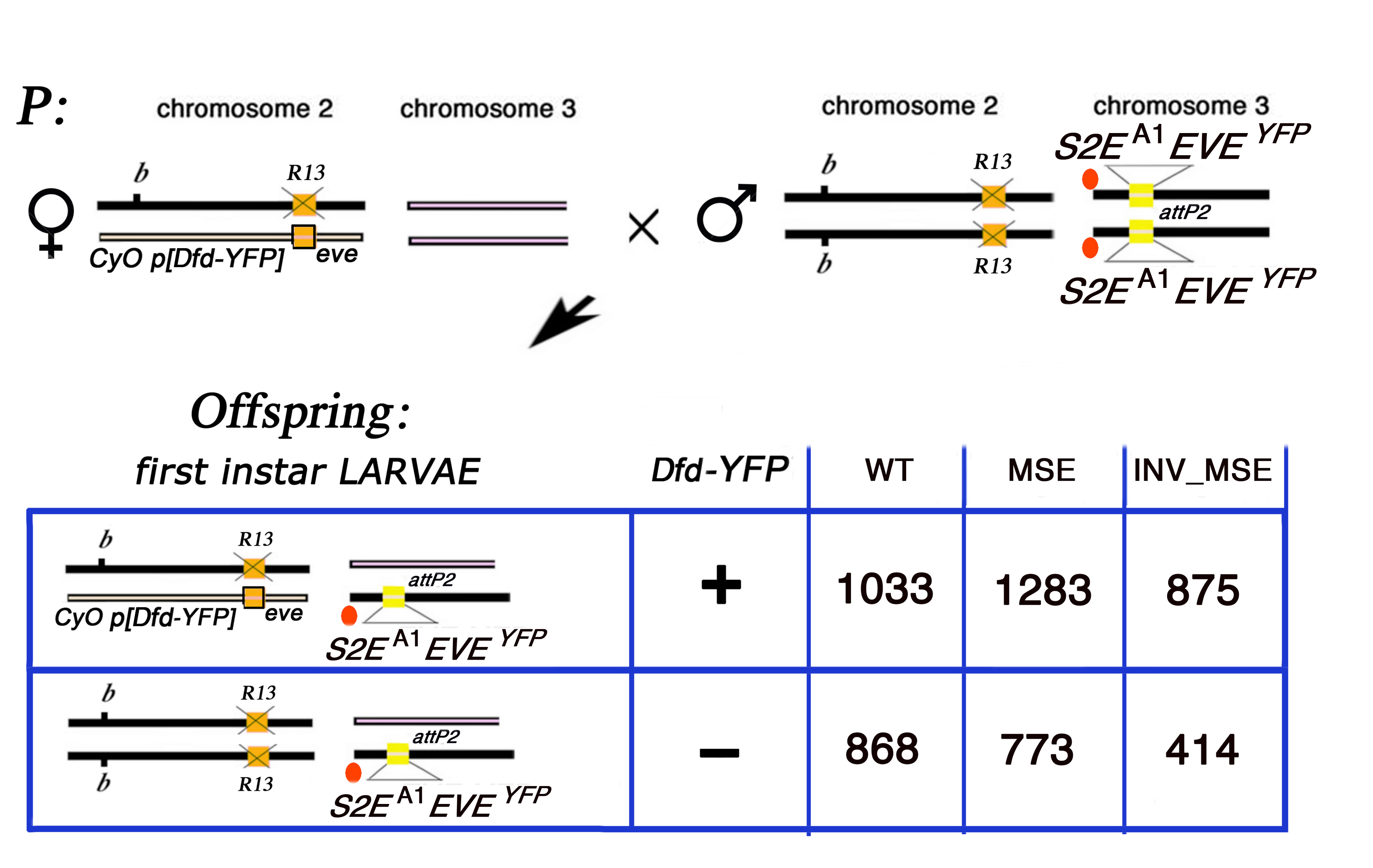

Supplement: Figure S4 — Viability of first instar larvae in the hemizygous rescue of the lethality of the R13 mutant. The crosses are as in Figure S1 with the exception that the second chromosome balancer has a P-element insertion of Deformed-YFP (Dfd-YFP) that allowed the scoring of larvae carrying the balancer. (TIF) [file pgen.1002364.s004.tif]

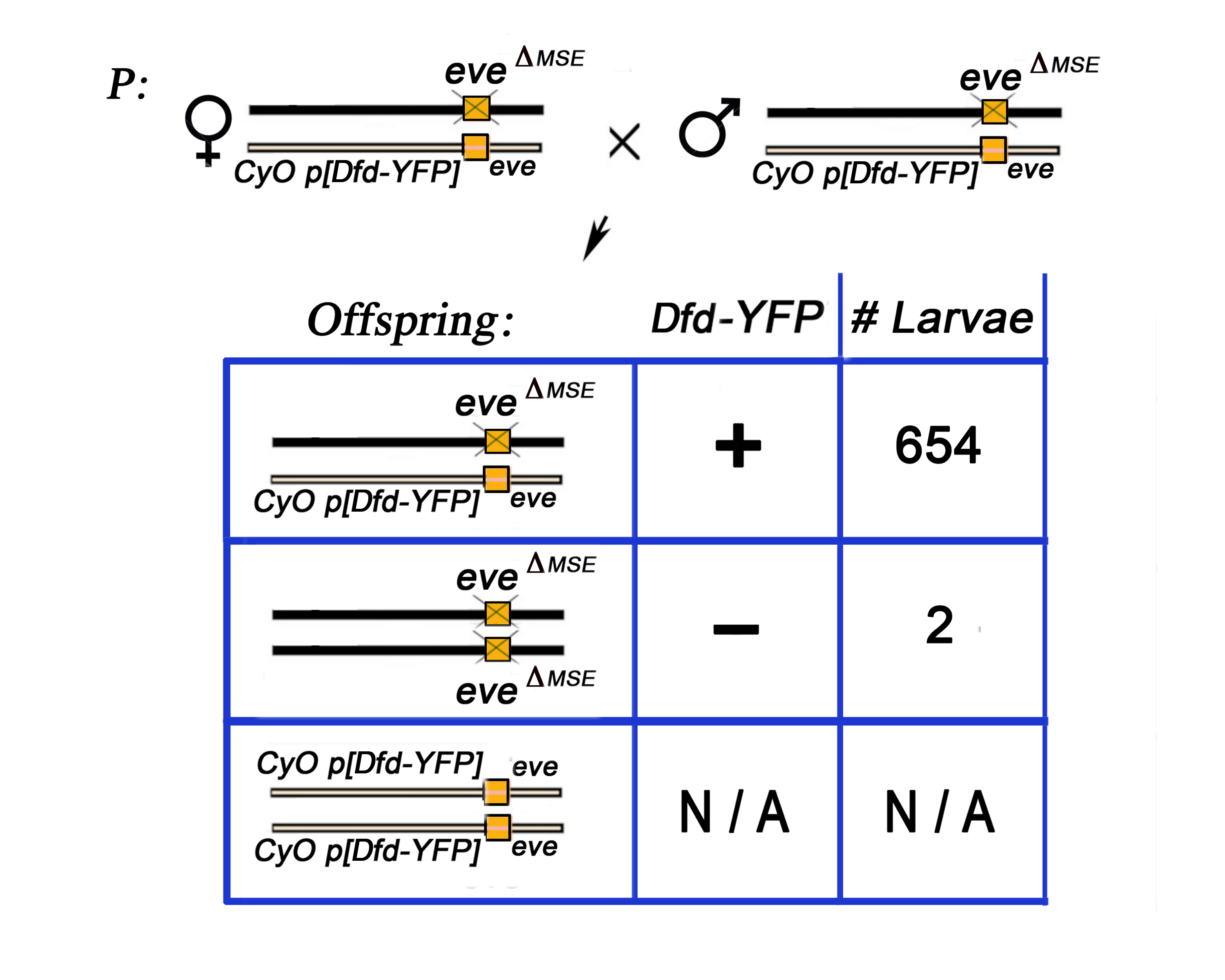

Supplement: Figure S5 — eveΔMSE causes embryonic lethality. The cross on top is between balanced eveΔMSE lines. The balancer carries a P-element insertion of Dfd-YFP. The offspring genotypes are in the first column, the second column indicates whether a genotype is expected to be Dfd-YFP positive or not and the third column has the number of hatched larvae counted. The third genotype is not observable because the CyO/CyO homozygote is embryonic lethal. (TIF) [file pgen.1002364.s005.tif]

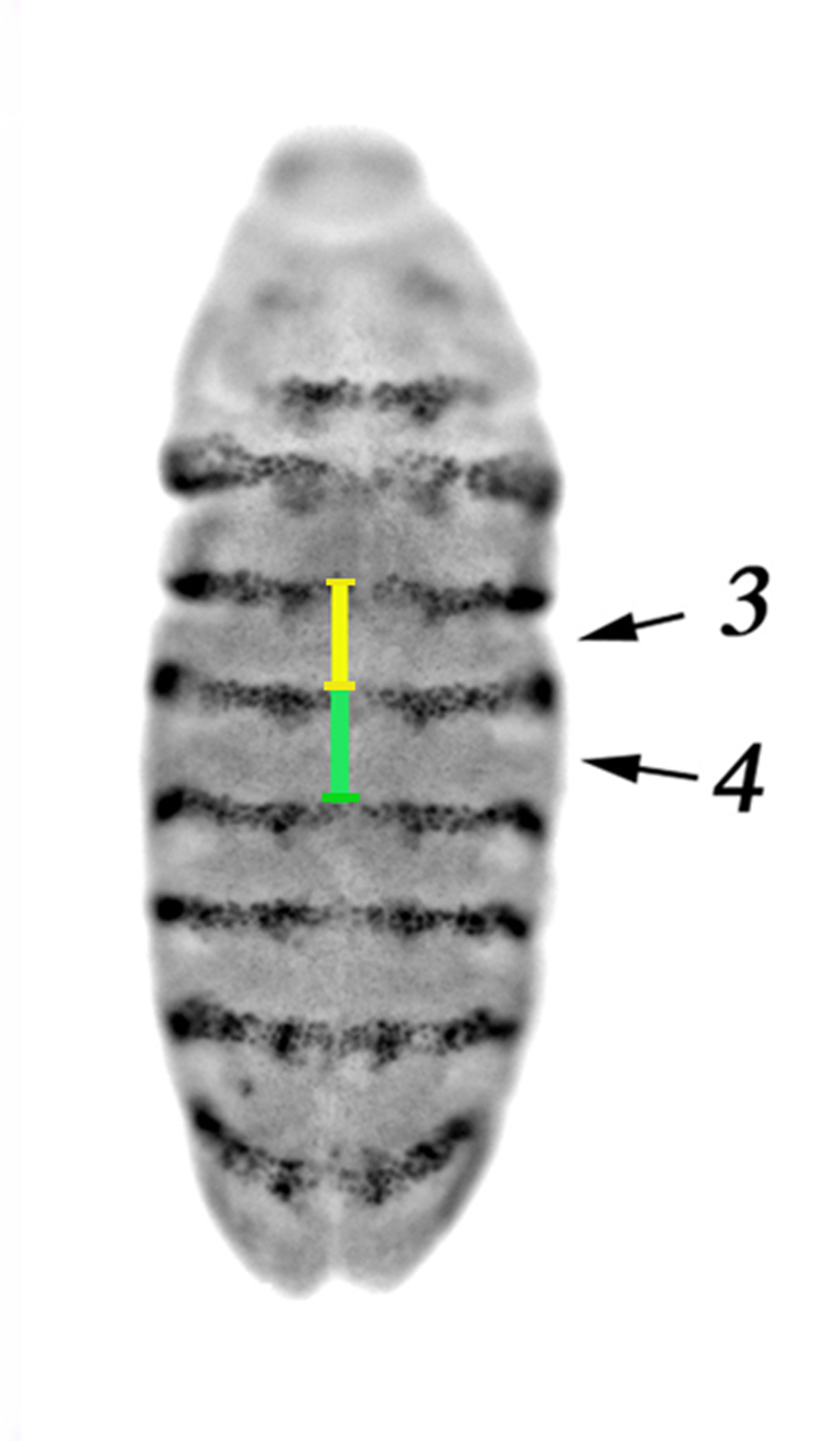

Supplement: Figure S6 — The lengths of parasegments 3 and 4 in the En pattern were measured at the ventral midline of embryos. Shown by green and yellow bars respectively. (TIF) [file pgen.1002364.s006.tif]

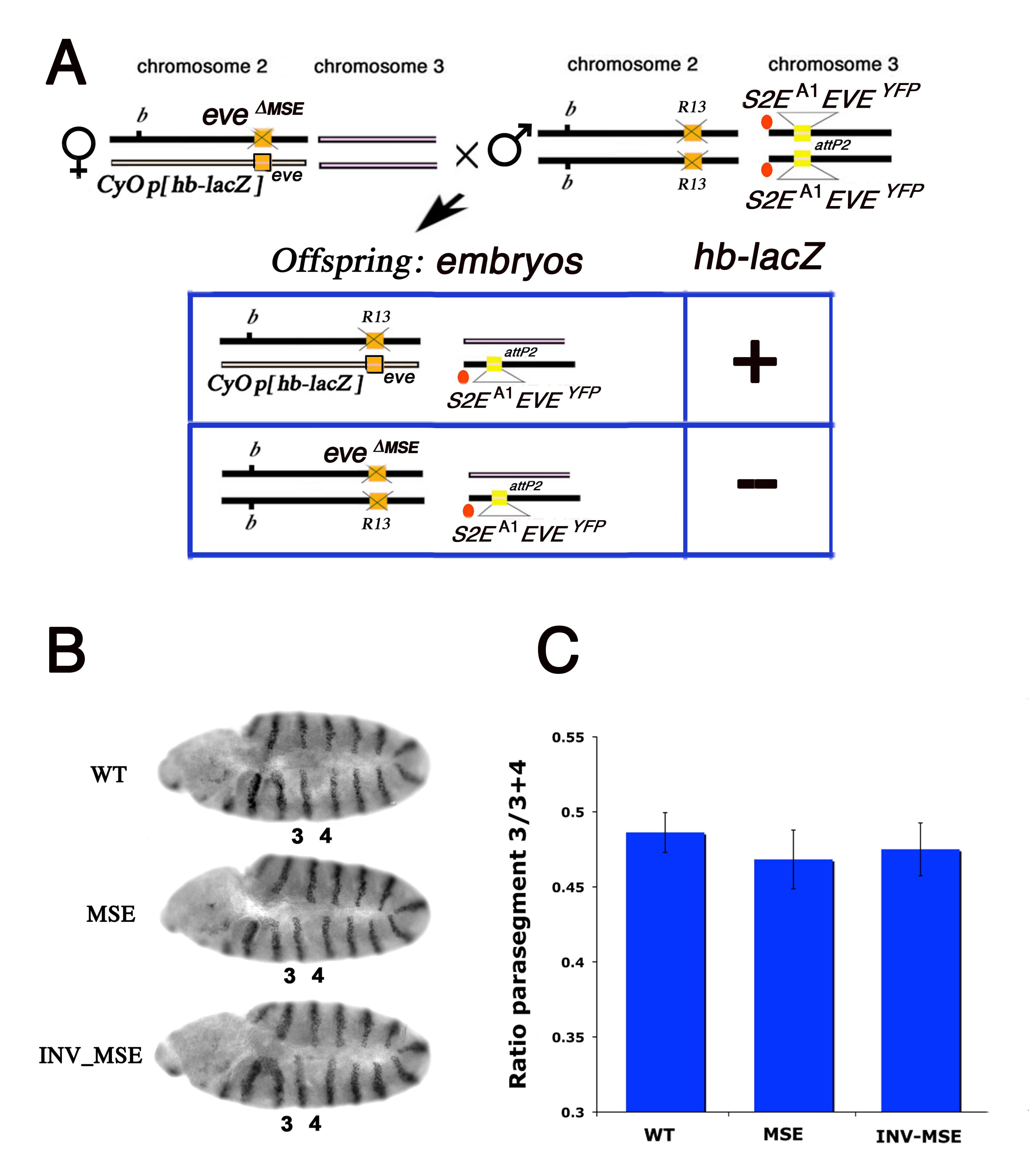

Supplement: Figure S7 — Parasegment 3 is reduced in the hemizygous rescue of eveΔMSE/R13 by the MSE or INV_MSE transgenes. A. Schema to study the En pattern in the eveΔMSE /R13 mutant embryos rescued by the altered eve transgenes. Example cross and relevant offspring genotypes for the assay. Genetic notation is the same as in Figure S1 with the exception that the second chromosome balancer has a P-element insertion of hb-lacZ that allowed the scoring of embryos carrying the balancer. The R13/eveΔMSE mutant embryos were identified by the absence of β-galactosidase expression and PCR genotyping. B. The En pattern in the R13/eveΔMSE mutant embryos with eve driven by one copy of the WT, MSE, or INV_MSE transgenes; stage 11. Note the variation in parasegments 3 and 4. C. Difference between WT and MSE (or INV_MSE) in En stripe 4 spatial expression was evaluated as the ratio of the length of parasegment 3 to the sum of parasegments 3+4 (see Methods and Figure S6). Error bars are standard deviations. N for each case is about 20. (TIF) [file pgen.1002364.s007.tif]

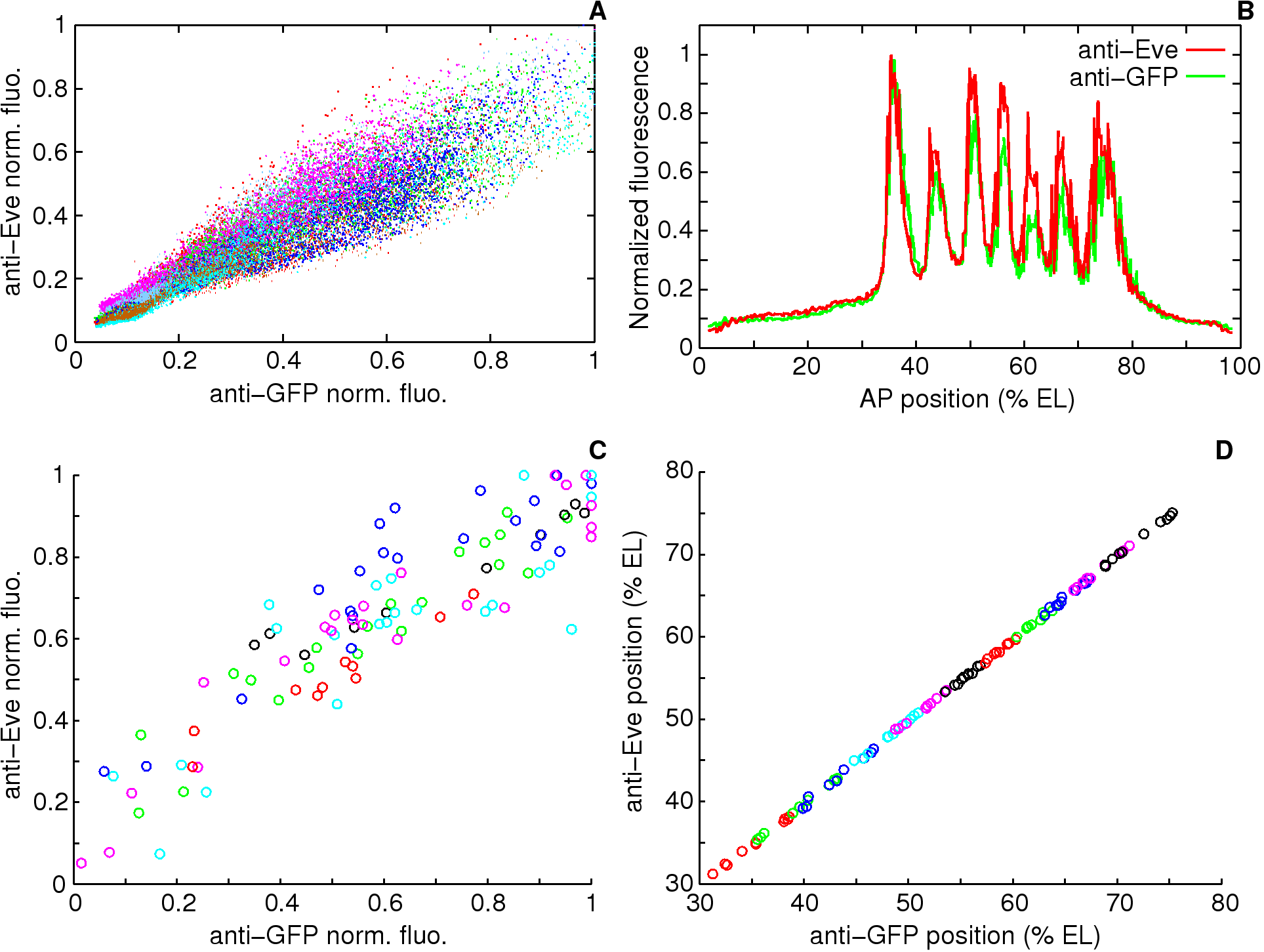

Supplement: Figure S8 — Eve-YFP faithfully reproduces endogenous Eve expression. Embryos carrying the Eve-YFP transgene were costained with anti-Eve and anti-GFP antibodies and imaged in a confocal microscope. The images were segmented (see Methods) and mean anti-Eve or anti-GFP fluorescence was calculated in each nucleus. A. A scatter plot of anti-Eve with anti-GFP fluorescence in all nuclei of 10 embryos. The data for each embryo are plotted with the same color. The values for each channel were normalized to maximum fluorescence observed in the embryo, but were not manipulated otherwise. The scatter shows strong proportionality as it lies along the diagonal (r2>0.88 for all embryos). B. anti-Eve and anti-GFP profiles extracted from a dorsoventral strip along the anteroposterior axis of the same embryo. Each profile is normalized to its maximum expression. The anti-GFP profile, mostly overlapping with the other, has lower expression in some stripes, most notably stripes five and six. Stripes 2-7 steadily increase expression during middle cycle 14 to achieve a level of expression equal to that of stripe one [35], with stripes five and six the last to reach maximum expression. The lower expression of the other stripes then suggests that the anti-GFP profile simply lags behind the anti-Eve profile. However, both profiles appear to belong to time class T6 of the staging scheme of Surkova et al. [36] implying that the lag is less than 6 min. C. Scatter plot of normalized border heights 2A-7A (see Methods). Border height eliminates the effect of background staining, since it is calculated as the difference in the expression of two closely spaced points in the embryo. Border heights also lie along the diagonal reflecting the proportionality of Eve-YFP expression to that of endogenous Eve. Border heights were extracted from the sample of embryos shown in panel A. N = 10. D. Scatter plot of stripe positions. r2>0.9998. Different positions are shown in different colors. Stripe positions were extrac [file pgen.1002364.s008.tif]

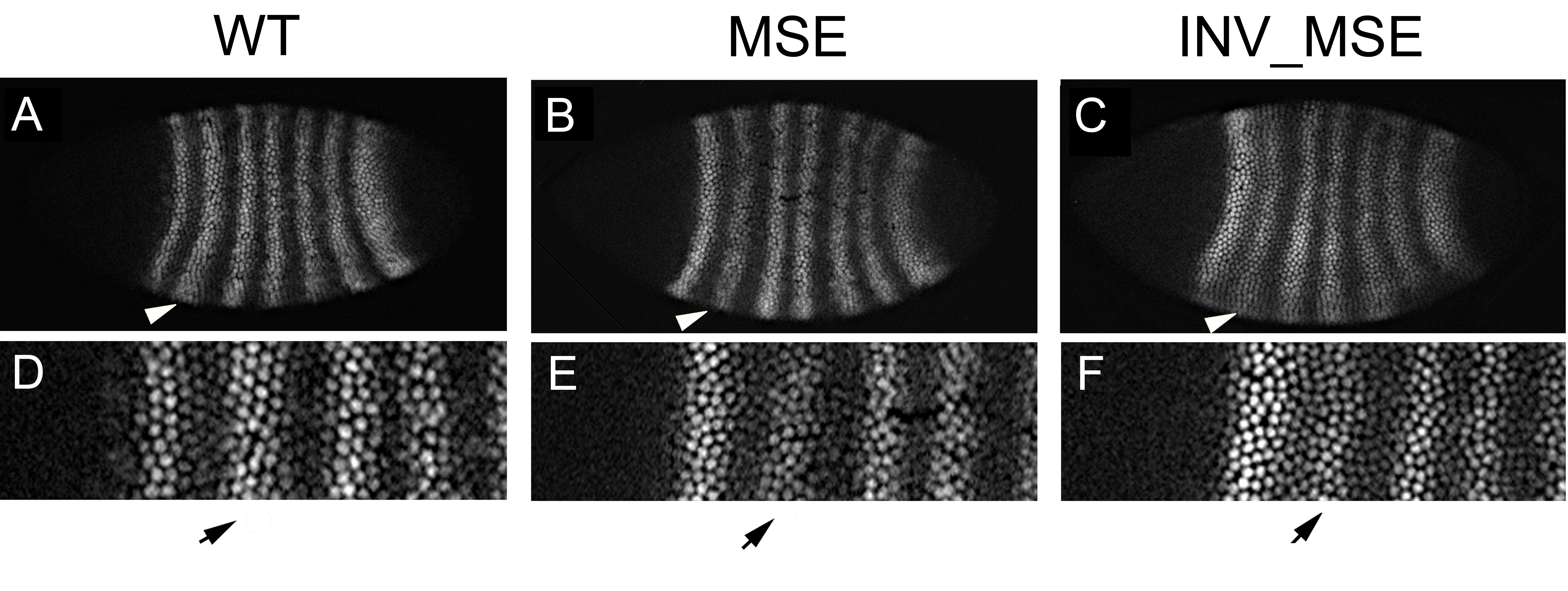

Supplement: Figure S9 — MSE and INV_MSE phenotypes in fixed tissue. Confocal images of mid cycle 14 embryos immunostained for YFP. A,D. WT, B,E. MSE, and C,F. INV_MSE. D-F. Magnified view of the anterolateral region. Arrows point to stripe 2. Stripe 2 expression is weaker in MSE and the anterior border is derepressed in INV_MSE, validating the phenotypes observed in live data. (TIF) [file pgen.1002364.s009.tif]

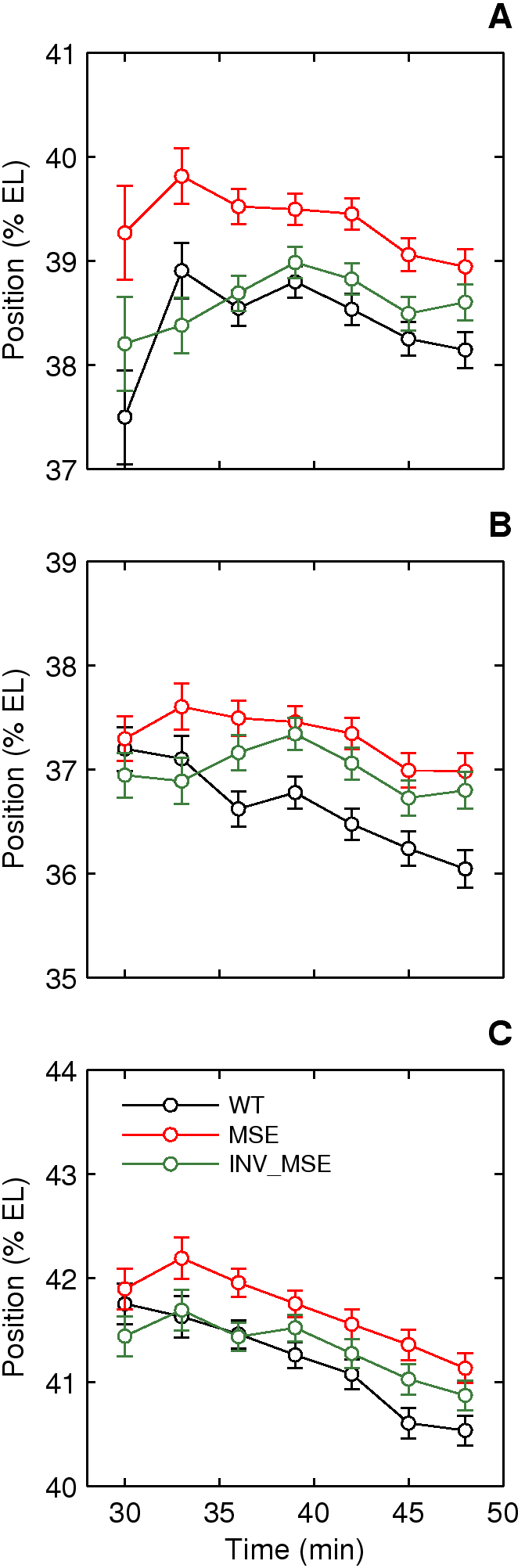

Supplement: Figure S10 — Positions of the peak and borders of stripe 2 do not differ between WT and MSE or INV_MSE. Plots and sample sizes are as in Figure 5. No statistically significant differences were observed (p>0.09 for all comparisons between WT and MSE or INV_MSE). A. Peak of stripe 2. B. Anterior border. C. Posterior border. (TIF) [file pgen.1002364.s010.tif]

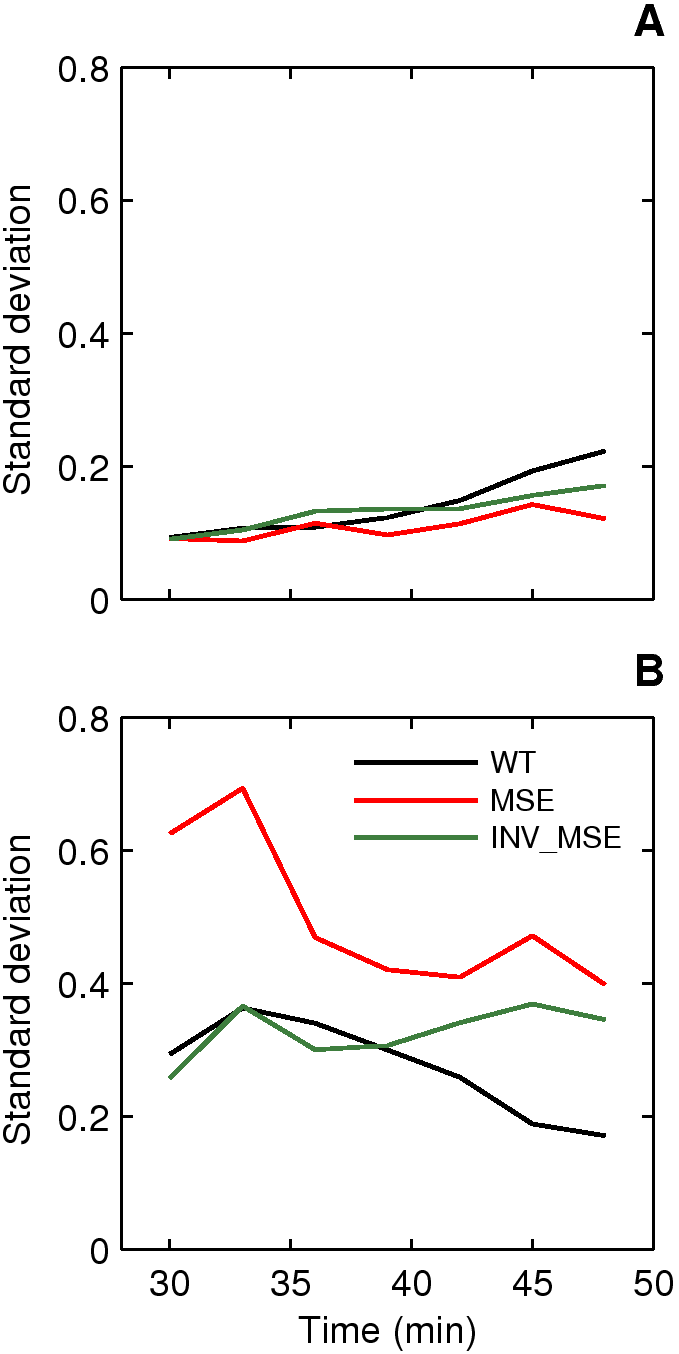

Supplement: Figure S11 — The variation of relative activation and repression of stripe 2 does not differ between WT and MSE or INV_MSE. Plots show time series of the standard deviation of the relative activation (A) and relative repression (B). Sample sizes are as in Figure 5. (TIF) [file pgen.1002364.s011.tif]

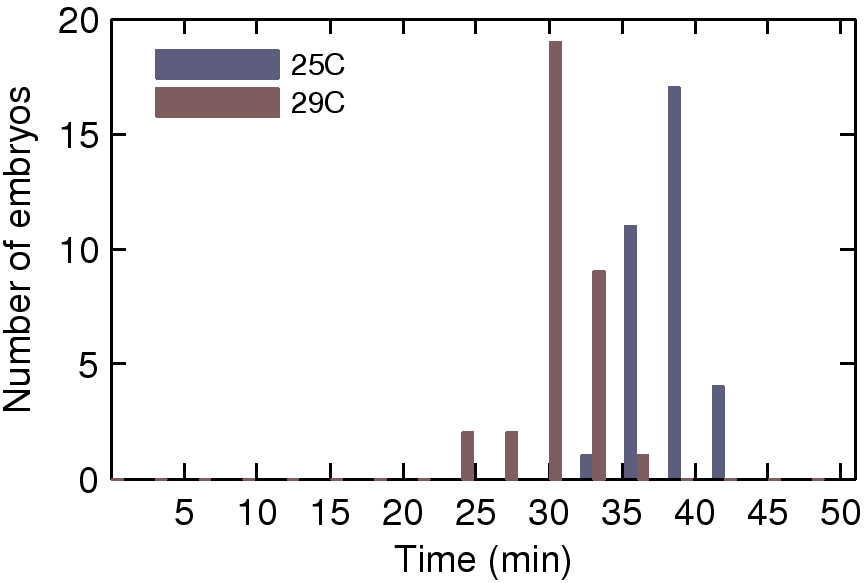

Supplement: Figure S12 — Accelerated rate of development at 29C. Using the histone channel of embryo movies (see Methods), we noted the time during cellularization when the cell membrane is at the basal end of nuclei [36], an easily identifiable morphological mark. The histograms of these times are shown for embryos developing at either 25C or 29C. The membrane reaches the basal end of nuclei at 38.2 (±2.2) min at 25C and at 30.6 (±2.5) min at 29C. (TIF) [file pgen.1002364.s012.tif]

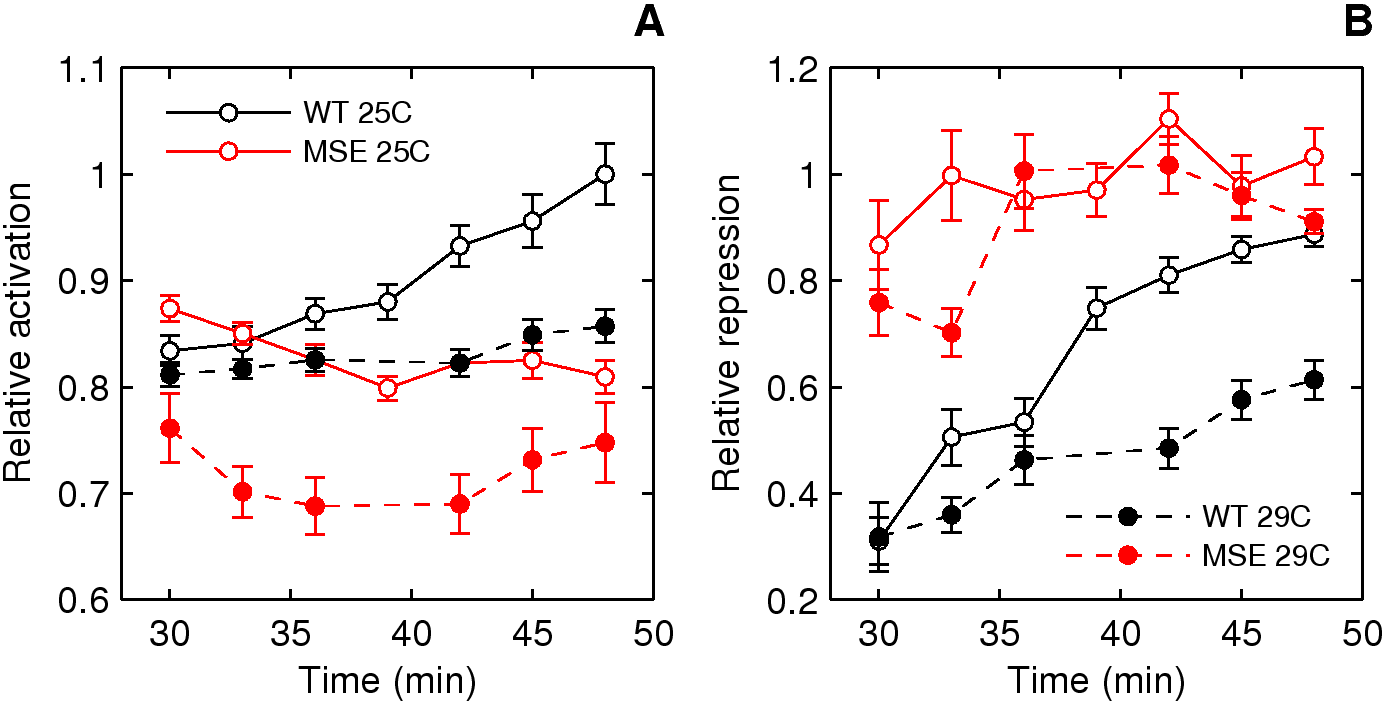

Supplement: Figure S13 — The effect of scaling time according to a developmental mark on the relative activation and repression of stripe 2. We plot the 29C data (Figure 9) at timepoints that are the product of absolute time with the ratio of the developmental rate at 29C to the rate at 25C (Figure S12). Our conclusions about the differential effect of temperature on these phenotypes are robust to such scaling. The relative activation of MSE at 29C still follows a progression that is qualitatively and quantitatively different from the one at 25C. However, the interpretation of the effect of temperature on WT differs. In absolute time, the phenotypes appear to follow the same trajectories at 29C as 25C except for a lag whereas, scaled to developmental time, there appears to be a deficit in the relative activation and repression of stripe 2. A. Relative activation. B. Relative repression. (TIF) [file pgen.1002364.s013.tif]

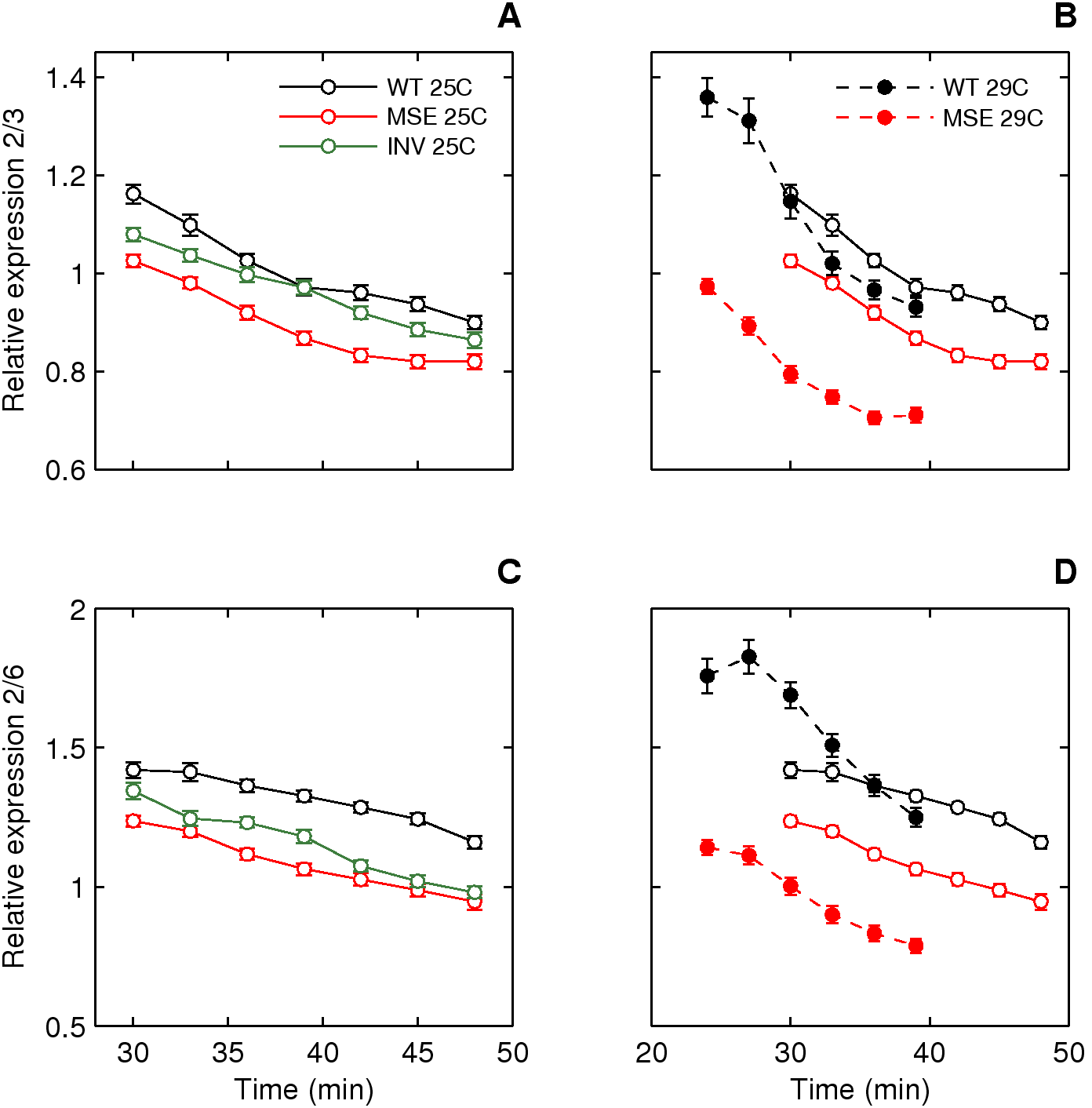

Supplement: Figure S14 — Time series of stripe 2 expression normalized by the expression of stripes 3 or 6. See Figure 5 and Figure 9 for an explanation of plots and sample sizes. A,B. Stripe 2 expression normalized to stripe 3 expression. C,D. Stripe 2 expression normalized to stripe 6 expression. A,C. Time series for 25C. Unlike stripe 1 expression, which is higher than stripe 2 throughout cycle 14, stripes 3 and 6 are lower than stripe 2 initially but increase in expression as the cycle progresses. As a result, stripe 2 expression normalized to these stripes displays a decreasing trend for WT instead of an increasing one (Figure 5E). However, normalized MSE expression is lower than WT and INV_MSE is intermediate, consistent with the phenotypes observed when normalizing with stripe 1. A. Between WT and MSE, p = 0.0143, 0.0188, 0.0150, 0.0275, 0.004, 0.0097, 0.089. Between WT and INV_MSE, p = 0.109, 0.1481, 0.6104, 0.9817, 0.4553, 0.229, 0.3947. C. Between WT and MSE, p = 0.0163, 0.0063, 0.001, 0.0009, 0.0007, 0.0008, 0.0062. Between WT and INV_MSE, p = 0.4757, 0.0464, 0.0308, 0.0628, 0.0014, 0.0016, 0.0121. B,D. Comparison of time series between 25C and 29C. As in Figure 9A, the time series at 25C and 29C overlap for WT, but not for MSE. B. For WT, between 25C and 29C, p = 0.3687, 0.1092, 0.1904, 0.253. For MSE, p = 0.0002, 3.1886e-05, 0.0001, 0.0019. D. For WT, p = 0.0149, 0.5308, 0.8843, 0.3496. For MSE, p = 0.014, 0.0007, 0.0011, 0.001. (TIF) [file pgen.1002364.s014.tif]
