# Supplementary material for: Consequences of Eukaryotic Enhancer Architecture for Gene Expression Dynamics, Development, and Fitness
Source: PLoS Genet. 2011 Nov 10;7(11):e1002364. doi: 10.1371/journal.pgen.1002364 (PMC3213169; doi:10.1371/journal.pgen.1002364)
Supplement: Table S1 — Adult survival and relative viability for the EVE transgene. (DOC) [file pgen.1002364.s015.doc]

**Table S1.** Adult survival and relative viability for the EVE transgene.

| **Sex** | **Genotype^a^** | **Observed** | **Expected**  **proportions** | **Relative Viability^b^ (%)** |
| --- | --- | --- | --- | --- |
| Female | CyO; TM3 | 250 | 4/9 | 103 |
| Female | CyO; N | 114 | 2/9 | 94 |
| Female | N; TM3 | 33 | 2/9 | 27 |
| Female | N; N | 52 | 1/9 | 86 |
| Male | CyO; TM3 | 192 | 4/9 | 103 |
| Male | CyO; N | 88 | 2/9 | 94 |
| Male | N; TM3 | 19 | 2/9 | 20 |
| Male | N; N | 38 | 1/9 | 81 |

**^a^** *CyO*: balancer second chromosome; *TM3*: balancer third chromosome; *N*: no balancer second or third chromosome.

**^b^** The observed ratios of the two genotypes carrying one copy of the endogenous *eve* locus (*CyO*) and either one (*TM3*) or two copies (N) of a transgene conformed to the expected 2:1 segregation ratio implying wildtype viabilities for both. On this basis, the numbers of adults produced by *CyO***;***TM3* and *CyO***;***N* genotypes were used to calculate the expected numbers for the rescue transgene genotypes (Figure S1; materials and methods).
