# Supplementary material for: Consequences of Eukaryotic Enhancer Architecture for Gene Expression Dynamics, Development, and Fitness
Source: PLoS Genet. 2011 Nov 10;7(11):e1002364. doi: 10.1371/journal.pgen.1002364 (PMC3213169; doi:10.1371/journal.pgen.1002364)
Supplement: Table S2 — Adult survival and relative viability of the WT, MSE, and INV_MSE transgenes. (DOC) [file pgen.1002364.s016.doc]

**Table S2.** Adult survival and relative viability of the WT, MSE, and INV_MSE transgenes**.**

| **Sex** | **Genotype^a^** | **Transgene** | | | **Relative Viability^b^** | | |
| --- | --- | --- | --- | --- | --- | --- | --- |
|  |  | **WT** | **MSE** | **INV_MSE** | **WT** | **MSE** | **INV_MSE** |
| Female | CyO; TM3 | 1299 | 1344 | 1215 | 100 | 103 | 98 |
| Female | CyO; N | 646 | 618 | 642 | 100 | 95 | 104 |
| Female | N; TM3 | 98 | 21 | 11 | 15 | 3.2 | 2 |
| Female | N; N | 215 | 249 | 272 | 66 | 76 | 88 |
| Male | CyO; TM3 | 1226 | 1141 | 1145 | 100 | 99 | 99 |
| Male | CyO; N | 621 | 585 | 598 | 101 | 102 | 103 |
| Male | N; TM3 | 71 | 8 | 5 | 12 | 1.4 | 1 |
| Male | N; N | 166 | 132 | 227 | 54 | 46 | 78 |

^a,b^ As in Table S1.
