# Supplementary material for: Consequences of Eukaryotic Enhancer Architecture for Gene Expression Dynamics, Development, and Fitness
Source: PLoS Genet. 2011 Nov 10;7(11):e1002364. doi: 10.1371/journal.pgen.1002364 (PMC3213169; doi:10.1371/journal.pgen.1002364)
Supplement: Table S3 — Ratio (X) of En parasegment 3 length relative to 3+4 in hemizygous embryos (Df(eve)/R13). (DOC) [file pgen.1002364.s017.doc]

**Table S3.** Ratio (*X*) of En parasegment 3 length relative to 3+4 in hemizygous embryos (*Df(eve)/*R13***).***

| **Stage** | **WT** | | | **MSE** | | | **INV_MSE** | | |
| --- | --- | --- | --- | --- | --- | --- | --- | --- | --- |
|  | ***N*** | ***X*(SE)** | ***N*** | | ***X*(SE)** | ***N*** | | ***X*(SE)** |  |
| 10 | 16 | 0.46 (0.022) | 22 | | 0.43** (0.024) | 16 | | 0.43** (0.032) |  |
| 11 | 15 | 0.47 (0.020) | 14 | | 0.43** (0.024) | 17 | | 0.43* (0.029) |  |

**p < 0.01; **p<0.001*
