# Supplementary material for: Consequences of Eukaryotic Enhancer Architecture for Gene Expression Dynamics, Development, and Fitness
Source: PLoS Genet. 2011 Nov 10;7(11):e1002364. doi: 10.1371/journal.pgen.1002364 (PMC3213169; doi:10.1371/journal.pgen.1002364)
Supplement: Table S4 — Ratio (X) of En parasegment 3 length relative to 3+4 in hemizygous embryos (eve△MSE /R13). (DOC) [file pgen.1002364.s018.doc]

**Table S4**. Ratio (*X*) of En parasegment 3 length relative to 3+4 in hemizygous embryos (*eve^△MSE^* /R13*).*

| **Stage** | **WT** | | **MSE** | | **INV-MSE** | |
| --- | --- | --- | --- | --- | --- | --- |
|  | ***N*** | ***X*(SE)** | ***N*** | ***X*(SE)** | ***N*** | ***X*(SE)** |
| 11 | 21 | 0.49 (0.013) | 21 | 0.47** (0.019) | 21 | 0.48  (0.019) |

***p < 0.01*
