# Supplementary material for: Consequences of Eukaryotic Enhancer Architecture for Gene Expression Dynamics, Development, and Fitness
Source: PLoS Genet. 2011 Nov 10;7(11):e1002364. doi: 10.1371/journal.pgen.1002364 (PMC3213169; doi:10.1371/journal.pgen.1002364)
Supplement: Text S1 — Primers and sequences. (DOC) [file pgen.1002364.s019.doc]

**Primers and sequences**

**1 Primers**

**1.1 PCR primers for recombineering**

**Primers for the *eve –YFP* fusion**

Primer_eveCYFP_F:

TGATTGCGGAGCCCAAGCCGAAGCTCTTCAAGCCCTACAAGACTGAGGCGGATTATGATATTCCAACTACTGCAAGCATGGTGAGCAAGGGCGAG

Primer_eveCYFP_R:

CTTTTGGGGGAGCATGGGGGGGGGGGAGAGAGTGTGTGTGGATCGCGGGCTTACTTGTACAGCTCGTCCATGC

**Primers to clone from BAC to attB_3xP3_DsRed_P15A-amp**

eve_intF2

GTAAACATGTCCCTTTATCTGGCAACGCTGCTACCCATTAAATTTGAAATCAGGCATTCAAATATGTATCC

eve_intR2

TAATCGATATTTTCTAAGCCCAATCGATGAATGTCCGGTAACGCACATCACGTCGACGATGTAGGTCACG

**Primers to create MSE enhancer**

eve_MSE_F

GGCGAGTTAATGCCAATGCAAATTGCGGGCGCAATATAACCCAATAATTTGTTACCCGGTACTGCATAACAA

eve_MSE_R

AAAGTTTTCGGTTTCGTTGGGCAAAACATTTATTATGATGATATAATCATTTAATTGCGTTGCCTGGACCTG

**Primers to create INV_MSE enhancer**

eve_MSE_inF

AAAGTTTTCGGTTTCGTTGGGCAAAACATTTATTATGATGATATAATCATGTTACCCGGTACTGCATAACAA

eve_MSE_inR

GGCGAGTTAATGCCAATGCAAATTGCGGGCGCAATATAACCCAATAATTTTTAATTGCGTTGCCTGGACCTG

**1.2 PCR Primers used to verify insertion of the vector attB into attP2 landing site**

attB-F GTCGACGATGTAGGTCACGGTC

Plac4 ACTGTGCGTTAGGTCCTGTTCATTGTT

attB-R TCGACATGCCCGCCGTGACCGTC

yellow49 GGCTTCACGTTTTCCCAGGTCAGAAGCGGT

**1.3 PCR Primers for genotyping the *P[hb-lacZ]* marker (long 800 bp) for balancer second chromosome**

Z353 CTGCCAGTTTGAGGGGACGACGACA

hb32 ACCAACGTAATCCCCATAGAAAA

**PCR Primers for positive marker (80bp) of PCR genotyping reaction**

sna-F CCCACGTGGACGTCAAGAA

sna-R GAGCGACATCCTGGAGAAAGA

**2 Sequences**

**2.1 Vector for the integration into attP2 docking site**

**attB_3xP3_DsRed_P15A-Amp^R^**  ( 3265 bp)

**The vector features:**

**7 - 290 attB site**

**297 – 579 3xP3 promoter for Pax-6**

**580 - 1259 DsRed**

**1267- 1504 SV40 polyA**

**1524 -2324 p15A ori**

**2325 -3185 Amp^R^**

***Italic* *font* - Restriction sites**

*GCTAGC***gtcgacgatgtaggtcacggtctcgaagccgcggtgcgggtgccagggcgtgcccttgggctccccgggcgcgtactccacctcacccatctggtccatcatgatgaacgggtcgaggtggcggtagttgatcccggcgaacgcgcggcgcaccgggaagccctcgccctcgaaaccgctgggcgcggtggtcacggtgagcacgggacgtgcgacggcgtcggcgggtgcggatacgcggggcagcgtcagcgggttctcgacggtcacggcgggcatgtcga***AGATCA***CAATGGTTAATTCGAGCTCGCCCGGGGATCTAATTCAATTAGAGACTAATTCAATTAGAGCTAATTCAATTAGGATCCAAGCTTATCGATTTCGAACCCTCGACCGCCGGAGTATAAATAGAGGCGCTTCGTCTACGGAGCGACAATTCAATTCAAACAAGCAAAGTGAACACGTCGCTAAGCGAAAGCTAAGCAAATAAACAAGCGCAGCTGAACAAGCTAAACAATCGGGGTACCGCTAGAGTCGACGGTACCGCGGGCCCGGGATCCACCGGTCGCCACCATGGTGCGCTCCTCCAAGAACGTCATCAAGGAGTTCATGCGCTTCAAGGTGCGCATGGAGGGCACCGTGAACGGCCACGAGTTCGAGATCGAGGGCGAGGGCGAGGGCCGCCCCTACGAGGGCCACAACACCGTGAAGCTGAAGGTGACCAAGGGCGGCCCCCTGCCCTTCGCCTGGGACATCCTGTCCCCCCAGTTCCAGTACGGCTCCAAGGTGTACGTGAAGCACCCCGCCGACATCCCCGACTACAAGAAGCTGTCCTTCCCCGAGGGCTTCAAGTGGGAGCGCGTGATGAACTTCGAGGACGGCGGCGTGGTGACCGTGACCCAGGACTCCTCCCTGCAGGACGGCTGCTTCATCTACAAGGTGAAGTTCATCGGCGTGAACTTCCCCTCCGACGGCCCCGTAATGCAGAAGAAGACCATGGGCTGGGAGGCCTCCACCGAGCGCCTGTACCCCCGCGACGGCGTGCTGAAGGGCGAGATCCACAAGGCCCTGAAGCTGAAGGACGGCGGCCACTACCTGGTGGAGTTCAAGTCCATCTACATGGCCAAGAAGCCCGTGCAGCTGCCCGGCTACTACTACGTGGACTCCAAGCTGGACATCACCTCCCACAACGAGGACTACACCATCGTGGAGCAGTACGAGCGCACCGAGGGCCGCCACCACCTGTTCCTGTA***GCGGCCGC***GACTCTAGATCATAATCAGCCATACCACATTTGTAGAGGTTTTACTTGCTTTAAAAAACCTCCCACACCTCCCCCTGAACCTGAAACATAAAATGAATGCAATTGTTGTTGTTAACTTGTTTATTGCAGCTTATAATGGTTACAAATAAAGCAATAGCATCACAAATTTCACAAATAAAGCATTTTTTTCACTGCATTCTAGTTGTGGTTTGTCCAAACTCATCAATGTAT*CTTAAG****CTCGAGGCGCGCCGAATTC***gcggaaatggcttacgaacggggcggagatttcctggaagatgccaggaagatacttaacagggaagtgagagggccgcggcaaagccgtttttccataggctccgcccccctgacaagcatcacgaaatctgacgctcaaatcagtggtggcgaaacccgacaggactataaagataccaggcgtttccccctggcggctccctcgtgcgctctcctgttcctgcctttcggtttaccggtgtcattccgctgttatggccgcgtttgtctcattccacgcctgacactcagttccgggtaggcagttcgctccaagctggactgtatgcacgaaccccccgttcagtccgaccgctgcgccttatccggtaactatcgtcttgagtccaacccggaaagacatgcaaaagcaccactggcagcagccactggtaattgatttagaggagttagtcttgaagtcatgcgccggttaaggctaaactgaaaggacaagttttggtgactgcgctcctccaagccagttacctcggttcaaagagttggtagctcagagaaccttcgaaaaaccgccctgcaaggcggttttttcgttttcagagcaagagattacgcgcagaccaaaacgatctcaagaagatcatcttattaaggggtctgacgctcagtggaacgaaaactcacgttaagggattttggtcatgagattatcaaaaaggatcttcacctagatccttttaaattaaaaatgaagttttaaatcaatctaaagtatatatgagtaaacttggtctgacagttaccaatgcttaatcagtgaggcacctatctcagcgatctgtctatttcgttcatccatagttgcctgactccccgtcgtgtagataactacgatacgggagggcttaccatctggccccagtgctgcaatgataccgcgagacccacgctcaccggctccagatttatcagcaataaaccagccagccggaagggccgagcgcagaagtggtcctgcaactttatccgcctccatccagtctattaattgttgccgggaagctagagtaagtagttcgccagttaatagtttgcgcaacgttgttgccattgctgcaggcatcgtggtgtcacgctcgtcgtttggtatggcttcattcagctccggttcccaacgatcaaggcgagttacatgatcccccatgttgtgcaaaaaagcggttagctccttcggtcctccgatcgttgtcagaagtaagttggccgcagtgttatcactcatggttatggcagcactgcataattctcttactgtcatgccatccgtaagatgcttttctgtgactggtgagtactcaaccaagtcattctgagaatagtgtatgcggcgaccgagttgctcttgcccggcgtcaacacgggataataccgcgccacatagcagaactttaaaagtgctcatcattggaaaacgttcttcggggcgaaaactctcaaggatcttaccgctgttgagatccagttcgatgtaacccactcgtgcacccaactgatcttcagcatcttttactttcaccagcgtttctgggtgagcaaaaacaggaaggcaaaatgccgcaaaaaagggaataagggcgacacggaaatgttgaatactcat**actcttcctttttcaatattattgaagcatttatcagggttattgtctcatgagcggatacatatttgaatg*CCTGCAGG*

**2.2 Sequences of eve stripe 2 enhancer regions**

All S2E, MSE, and INV-MSE enhancers are bordered on the 3′ and 5′ sides by completely conserved blocks of 18 bp and 26 bp, respectively (marked by bold font; also marked as blocks **a** and **b** in Figure 1)

**Wild type S2E: 798bp**

**AATATAACCCAATAATTT**GAAGTAACTGGCAGGAGCGAGGTATCCTTCCTGGTTACCCGGTACTGCATAACAATGGAACCCGAACCGTAACTGGGACAGATCGAAAAGCTGGCCTGGTTTCTCGCTGTGTGTGCCGTGTTAATCCGTTTGCCATCAGCGAGATTATTAGTCAATTGCAGTTGCAGCGTTTCGCTTTCGTCCTCGTTTCACTTTCGAGTTAGACTTTATTGCAGCATCTTGAACAATCGTCGCAGTTTGGTAACACGCTGTGCCATACTTTCATTTAGACGGAATCGAGGGACCCTGGACTATAATCGCACAACGAGACCGGGTTGCGAAGTCAGGGCATTCCGCCGATCTAGCCATCGCCATCTTCTGCGGGCGTTTGTTTGTTTGTTTGCTGGGATTAGCCAAGGGCTTGACTTGGAATCCAATCCCGATCCCTAGCCCGATCCCAATCCCAATCCCAATCCCTTGTCCTTTTCATTAGAAAGTCATAAAAACACATAATAATGATGTCGAAGGGATTAGGGGCGCGCAGGTCCAGGCAACGCAATTAACGGACTAGCGAACTGGGTTATTTTTTTGCGCCGACTTAGCCCTGATCCGCGAGCTTAACCCGTTTTGAGCCGGGCAGCAGGTAGTTGTGGGTGGACCCCACGATTTTTTTGGCCAAACCTCCAAGCTAACTTGCGCAAGTGGCAAGTGGCCGGTTTGCTGGCCCAAAAGAGGAGGCACTATCCCGGTCCTGGTACAGTTGGTACGCTGGGAA**TGATTATATCATCATAATAAATGTTT**

**MSE: 554 bp**

**AATATAACCCAATAATTT**GTTACCCGGTACTGCATAACAATGGAACCCGAACCGTAACTGGGACAGATCGAAAAGCTGGCCTGGTTTCTCGCTGTGTGTGCCGTGTTAATCCGTTTGCCATCAGCGAGATTATTAGTCAATTGCAGTTGCAGCGTTTCGCTTTCGTCCTCGTTTCACTTTCGAGTTAGACTTTATTGCAGCATCTTGAACAATCGTCGCAGTTTGGTAACACGCTGTGCCATACTTTCATTTAGACGGAATCGAGGGACCCTGGACTATAATCGCACAACGAGACCGGGTTGCGAAGTCAGGGCATTCCGCCGATCTAGCCATCGCCATCTTCTGCGGGCGTTTGTTTGTTTGTTTGCTGGGATTAGCCAAGGGCTTGACTTGGAATCCAATCCCGATCCCTAGCCCGATCCCAATCCCAATCCCAATCCCTTGTCCTTTTCATTAGAAAGTCATAAAAACACATAATAATGATGTCGAAGGGATTAGGGGCGCGCAGGTCCAGGCAACGCAATTAAA**TGATTATATCATCATAATAAATGTTT**

**INV_MSE: 554 bp**

**AATATAACCCAATAATTT**TTAATTGCGTTGCCTGGACCTGCGCGCCCCTAATCCCTTCGACATCATTATTATGTGTTTTTATGACTTTCTAATGAAAAGGACAAGGGATTGGGATTGGGATTGGGATCGGGCTAGGGATCGGGATTGGATTCCAAGTCAAGCCCTTGGCTAATCCCAGCAAACAAACAAACAAACGCCCGCAGAAGATGGCGATGGCTAGATCGGCGGAATGCCCTGACTTCGCAACCCGGTCTCGTTGTGCGATTATAGTCCAGGGTCCCTCGATTCCGTCTAAATGAAAGTATGGCACAGCGTGTTACCAAACTGCGACGATTGTTCAAGATGCTGCAATAAAGTCTAACTCGAAAGTGAAACGAGGACGAAAGCGAAACGCTGCAACTGCAATTGACTAATAATCTCGCTGATGGCAAACGGATTAACACGGCACACACAGCGAGAAACCAGGCCAGCTTTTCGATCTGTCCCAGTTACGGTTCGGGTTCCATTGTTATGCAGTACCGGGTAACA**TGATTATATCATCATAATAAATGTTT**

**2.3 Sequence of SYFP2**

ATGGTGAGCAAGGGCGAGGAGCTGTTCACCGGGGTGGTGCCCATCCTGGTCGAGCTGGACGGCGACGTAAACGGCCACAAGTTCAGCGTGCGCGGCGAGGGCGAGGGCGATGCCACCAACGGCAAGCTGACCCTGAAGCTGATCTGCACCACCGGCAAGCTGCCCGTGCCCTGGCCCACCCTCGTGACCACCCTGGGCTACGGCGTGCAGTGCTTCGCCCGCTACCCCGACCACATGAAGCAGCACGACTTCTTCAAGTCCGCCATGCCCGAAGGCTACGTCCAGGAGCGCACCATCTTCTTCAAGGACGACGGCACCTACAAGACCCGCGCCGAGGTGAAGTTCGAGGGCGACACCCTGGTGAACCGCATCGAGCTGAAGGGCATCGACTTCAAGGAGGACGGCAACATCCTGGGGCACAAGCTGGAGTACAACTTCAACAGCCACAACGTCTATATCACCGCCGACAAGCAGAAGAACGGCATCAAGGCCAACTTCAAGATCCGCCACAACGTGGAGGACGGCGGCGTGCAGCTCGCCGACCACTACCAGCAGAACACCCCCATCGGCGACGGCCCCGTGCTGCTGCCCGACAACCACTACCTGAGCTACCAGTCCAAGCTGAGCAAAGACCCCAACGAGAAGCGCGATCACATGGTCCTGCTGGAGTTCGTGACCGCCGCCGGGATCACTCACGGCATGGACGAGCTGTACAAGTAA

**2.4 Sequence of genomic eve -6.6 to +9.8: 16 380 bp**

*Drosophila melanogaster* Reference Sequence Release 5.30

Start 5860288

End 5876667
